# Extracellular vesicles derived from *Enterococcus faecalis*: inflammatory activation does not require internalization

**DOI:** 10.1186/s12964-026-02926-9

**Published:** 2026-05-18

**Authors:** Marlon Alexander Gancino Guevara, Arefeh Kardani, Annika Schomisch, Sari Rasheed, Vida Mashayekhi, Emely Saccon, Nurzhan Abdukarimov, Nikolay Krasimirov Kirilov, Sabryna Junker, Agnes-Valencia Weiss, Marcus Koch, Gilles Gasparoni, Marc Schneider, Julia Schulze-Hentrich, Markus Bischoff, Sören L. Becker, Rolf Müller, Daniela Yildiz, Gregor Fuhrmann, Oskar Staufer, Jessica Hoppstädter, Alexandra K. Kiemer

**Affiliations:** 1 Department of Pharmacy, Pharmaceutical Biology, Saarland University, Saarbrücken, 66123 Germany; 2 Helmholtz Institute for Pharmaceutical Research Saarland (HIPS) ‑ Helmholtz Centre for Infection Research (HZI), and Department of Pharmacy, Saarland University, Saarbrücken, 66123 Germany; 3 German Centre for Infection Research (DZIF), Partner Site Hannover-Braunschweig, Braunschweig, 38124 Germany; 4 PharmaScienceHub (PSH), Saarbrücken, 66123 Germany; 5 INM – Leibniz Institute for New Materials, Saarbrücken, 66123 Germany; 6 Center for Biophysics, Saarland University, Saarbrücken, 66123 Germany; 7 Institute of Medical Microbiology and Hygiene, Saarland University, Homburg, 66421 Germany; 8 Department of Pharmacy, Biopharmaceutics and Pharmaceutical Technology, Saarland University, Saarbrücken, 66123 Germany; 9 Institute for Physical Process Technology, Saarland University of Applied Sciences, Saarbrücken, 66117 Germany; 10 Department of Genetics/Epigenetics, Saarland University, Saarbrücken, 66123 Germany; 11 Institute for Experimental and Clinical Pharmacology and Toxicology, Center for Molecular Signaling (PZMS), Saarland University, Homburg, 66421 Germany; 12 Center for Gender-Specific Biology and Medicine (CGBM), Saarland University, Homburg, 66421 Germany; 13 Chair of Pharmaceutical Biology, Department of Biology, Faculty of Science, Friedrich-Alexander Universität Erlangen-Nürnberg, Erlangen, 91058 Germany; 14 FAU Research Center New Bioactive Compounds (FAU NeW), Friedrich-Alexander-Universität Erlangen-Nürnberg, Erlangen, 91058 Germany

**Keywords:** Gram-positive bacterial EVs, NF-κB, TLR2, Pam_3_CSK_4_, Small unilamellar vesicles, Dynamin-dependent endocytosis, *ex vivo* embryonic zebrafish macrophages, HMDMs, HUVECs, extracellular flux analysis

## Abstract

**Background:**

*Enterococcus faecalis* is a common gut commensal Gram-positive bacterium that can act as an opportunistic pathogen and is frequently associated with severe infections community-acquired and nosocomial. Bacteria-derived extracellular vesicles (EVs) emerge as key mediators of host-bacteria communication with immunomodulatory roles and mechanistic participation in pathophysiological processes. However, the impact of *E. faecalis*-derived EVs (*Ef*-EVs) on host cells and their potential role in shaping host responses during infection remain unclear.

**Methods:**

*Ef*-EVs from the *E. faecalis* DSM 20478 type strain and four independent clinical bloodstream isolates were isolated *via* ultracentrifugation and size exclusion chromatography. EVs were characterized by nanoparticle tracking analysis and cryogenic transmission electron microscopy. Immunomodulatory effects of *Ef*-EVs were studied in vitro on NF-κB/AP-1 reporter cells, primary human monocyte-derived macrophages, and human umbilical vein endothelial cells, and by transcriptomic analysis of macrophages isolated from in vivo EV-treated zebrafish larvae. EV-induced signaling mechanisms were studied using uptake inhibitors as well as bottom-up assembled bacterial EVs functionalized with synthetic bacterial ligands. EV-induced metabolic reprogramming in macrophages was investigated by RNA-Seq and live-cell metabolic analyses using the Seahorse XFe-96 Flux Analyzer.

**Results:**

We found that *Ef*-EVs can induce pro-inflammatory responses in host macrophages *via* Toll-like receptor 2 (TLR2) signaling, as demonstrated using TLR2 transgenic cell lines and a TLR2-blocking antibody. Using uptake inhibitors as well as bottom-up assembled bacterial EVs functionalized with synthetic bacterial ligands as a minimalistic approach to study mechanisms of EV signaling, we demonstrated that *Ef*-EVs target the plasma membrane TLR2 to induce inflammation in a process uncoupled from their internalization. Furthermore, we found that *Ef*-EVs induce metabolic reprogramming towards a pro-inflammatory, glycolytic phenotype.

**Conclusion:**

Our findings reveal a mechanism by which Gram-positive bacterial EVs modulate immune signaling and metabolic pathways, advancing our understanding of host-pathogen communication.

**Supplementary Information:**

The online version contains supplementary material available at 10.1186/s12964-026-02926-9.

## Abstract (plain language summary)

*Enterococcus faecalis* is a commensal bacterium in the human gut, but it can also cause life-threatening diseases, especially serious hospital-acquired infections. Bacteria release particles called extracellular vesicles (EVs), which help them interact and communicate with other cells, including bacteria and human cells. However, it is unclear how EVs produced by *E. faecalis* (*Ef*-EVs) affect the host immune system. In this study, we investigated how *Ef*-EVs affect immune and endothelial cells. We found that *Ef*-EVs activate inflammatory responses through a receptor on the cell surface, without the need to be taken up by the cells. In addition, *Ef*-EVs altered the metabolism of immune cells, shifting them towards a state that supports inflammation. These findings highlight a previously underexplored mechanism by which Gram-positive bacterial EVs can shape host immunity and cellular metabolism, thereby advancing our understanding of host-pathogen interactions.

**Supplementary Information:**

The online version contains supplementary material available at 10.1186/s12964-026-02926-9.

## Background

*Enterococcus faecalis* is a Gram-positive bacterium and a commensal member of the human gut microbiota [1]. However, it can also act as an opportunistic pathogen capable of causing a range of life-threatening diseases, including peritonitis [1], bacteremia [2], endocarditis of both native and prosthetic heart valves [3], and sepsis [4], with a heightened pathogenicity in individuals with a compromised immune system or underlying health conditions [2]. Furthermore, *E. faecalis* is a major cause for nosocomial and iatrogenic infections, such as surgical site infections [5], catheter-associated urinary tract infections [6], and refractory periapical periodontitis [7]. Of note, the emergence of vancomycin-resistant enterococci represents also a major challenge for infection prevention and hospital hygiene at a global scale [8–10]. Upon transitioning from a commensal to a pathogenic bacterium, *E. faecalis* employs diverse mechanisms to interact with host cells and shape the course of infection, influencing not only colonization but also the host’s ability to mount an effective defense [1]. Indeed, the severity of enterococcal infections is often associated with the ability of enterococci to form biofilms [3, 11]. *E. faecalis* is also able to persist and replicate intracellularly in host cells [12], including epithelial cells [13], endothelial cells [14], and macrophages [12, 15, 16]. However, despite extensive characterization of the factors involved in *E. faecalis* pathogenicity, the mechanisms that facilitate bloodstream translocation, immune evasion, and survival, as well as virulence, remain to be further elucidated [3, 11, 17].

Beyond direct contact-based interactions, bacteria can remotely influence host cell responses through the release of extracellular vesicles (EVs) [18–21]. These nanosized membrane-bound particles serve as molecular messengers, carrying diverse bioactive cargo, including proteins, nucleic acids, and lipids [19, 21]. Upon contact with host cells, bacteria-derived EVs can modulate their function, initiating and shaping the course of pathophysiological processes, such as inflammation and infection [18, 22–24].

The formation and release of EVs is viewed as a secretory mechanism conserved across all domains of life (i.e., *Archaea*, *Bacteria*, and *Eukarya*) [19, 20, 25, 26]. Although the regulation of vesiculogenesis remains poorly understood [19, 26], studies indicate that EVs originate from the budding of membrane regions with tightly regulated lipid composition [19, 25].

Given the widespread assumption that the thick cell wall of Gram-positive bacteria interferes with EV biogenesis, research has historically focused on understanding the biological functions and the relevance of EVs derived from Gram-negative bacteria, leaving the roles of Gram-positive bacteria-derived EVs largely unexplored [20, 25, 27]. First identified by Lee et al. in *Staphylococcus aureus* [20], experimental evidence shows that Gram-positive bacteria-derived EVs shed from the bacterial cytoplasmic membrane, incorporating and transporting pathogen-associated molecular patterns (PAMPs), including lipoproteins, nucleic acids, and peptidoglycans (PGNs) [19, 20, 28]. As a source for bacterial antigens, Gram-positive bacterial EVs can be recognized by host pattern recognition receptors (PRRs), such as Toll-like receptors (TLRs) and nucleotide-binding oligomerization domain (NOD)-like receptors (NLRs), inducing signaling cascades that lead to the pro-inflammatory activation of immune cells [19, 28–30]. Studies on the signaling mechanisms induced by components derived from the cell membrane of Gram-positive bacteria (e.g., lipoteichoic acid (LTA) and acylated lipopeptides) indicate that induced cell activation is principally orchestrated by TLR2 [31] and suggest that their pro-inflammatory activity depends on their uptake following receptor binding [32]. Therefore, it raises the question of whether the immunomodulatory effects of Gram-positive bacterial EVs are functionally linked to their uptake.

During either exogenous or endogenous bacterial invasion, secreted bacterial EVs can permeate host barriers and access the host bloodstream [33–36]. Through complex interactions with host cells, involving multiple immune and non-immune cell types, bacteria-derived EVs may shape the outcome of bacterial infections and associated complications [33, 37]. In this context, the role of *E. faecalis*-derived EVs (*Ef*-EVs) as a virulence factor in *E. faecalis* pathogenicity during the progression of bloodstream infections (e.g., bacteremia, infective endocarditis, and sepsis) is still unclear [38, 39].

In this work, we investigated the immunomodulatory functions and induced signaling mechanisms of EVs derived from the *E. faecalis* DSM 20478 type strain and clinical bloodstream isolates in vitro using defined cellular model systems, such as NF-κB/AP-1 reporter cells, primary human monocyte-derived macrophages (HMDMs), and primary human umbilical vein endothelial cells (HUVECs). The in vivo effects of *Ef*-EVs were studied through transcriptomic analysis of e*x vivo* macrophages isolated from EV-treated zebrafish larvae – a relevant 3R-compatible model system. Leveraging synthetically bottom-up assembled bacterial EVs – i.e., small unilamellar vesicles (SUVs) precisely functionalized with the synthetic PRR ligand Pam_3_CSK_4_ – as a minimalistic model to study mechanisms of EV signaling [40], we found that *Ef*-EVs target the plasma membrane TLR2 to induce inflammation in a process uncoupled from their cellular internalization. Furthermore, we demonstrate that *Ef*-EVs trigger metabolic reprogramming towards a pro-inflammatory state. Taken together, these findings provide mechanistic insights into the role of *Ef*-EVs as a potential virulence factor and key mediator of host-pathogen interactions.

## Materials and methods

### Cell culture

#### Human primary cells

##### Human umbilical vein endothelial cells (HUVECs)

Human umbilical vein endothelial cells (HUVECs) were isolated from fresh umbilical cords from female individuals (Klinikum Saarbrücken, Germany, consent of the Local Ethics Committee, permission no. 131/08) under sterile conditions using 0.1 g/L collagenase for digestion (cat. no. COLLA-RO, Roche) at 37 °C. To stop the digestion, the veins were rinsed with Earle’s medium 199 (cat. no. P04-07500, PAA) containing 10% fetal calf serum (FCS, cat. no. F7524, PAA), 100 U/mL penicillin G, and 100 µg/mL streptomycin (cat. no. P4333, Sigma-Aldrich). After centrifugation (10 min, 200 × *g*), cells were re-suspended in 5 mL endothelial cell growth medium with supplement mix (cat. no. C-22010, Promocell) containing 10% FCS, 100 U/mL-100 µg/mL penicillin-streptomycin (Pen-Strep, cat. no. P4333, Sigma-Aldrich), and 0.1% Kanamycin (cat. no. K0254, Sigma-Aldrich), and cultivated at 37 °C and 5% CO_2_ in a T25 cell culture flask. After one day, the cells were washed three times with phosphate-buffered saline (PBS, 7.20 g/L NaCl, 0.43 g/L KH_2_PO_4_, 1.48 g/L Na_2_HPO_4_) and cultivated until they reached confluence. Cells were cryopreserved in passage #1 and were used for the experiments up to passage #6 [41].

##### Human monocyte-derived macrophages (HMDMs)

Monocytes were isolated from buffy coats of healthy blood donors (Blood Donation Center, Klinikum Saarbrücken, Germany) with the consent of the Local Ethics Committee (permission no. 173/18). Peripheral blood mononuclear cells (PBMCs) were isolated by density gradient centrifugation using lymphocyte separation medium 1077 (cat. no. C-44010, PromoCell) and LeucoSep tubes (cat. no. 227290, Greiner). Monocytes were obtained by magnetic cell sorting using anti-CD14 microbeads (cat. no. 130-050-201, Miltenyi). HMDMs were seeded and differentiated in RPMI 1640 medium (cat. no. R0883, Sigma-Aldrich) containing 2 mM L-glutamine, 1.5 g/L sodium bicarbonate, 4.5 g/L glucose, 10 mM HEPES, 1.0 mM sodium pyruvate, 10% FCS, 100 U/mL-100 µg/mL Pen-Strep, and 20 ng/mL human recombinant macrophage colony-stimulating factor (M-CSF, cat. no. 130-096-492, Miltenyi) for 6 days before their use [42].

#### NF-κB/AP-1 reporter cell lines

##### Differentiated macrophage-like THP1-XBlue™ cells

NF-κB/AP-1 reporter monocytes, THP1-XBlue™ cells (cat.no. thpx-sp, Invivogen), derived from the human monocytic THP-1 cell line by transfection of a secreted embryonic alkaline phosphatase (SEAP) reporter gene under the control of an NF-κB- and AP-1-inducible promoter, were cultured in RPMI 1640 medium containing 2 mM L-glutamine, 1.5 g/L sodium bicarbonate, 4.5 g/L glucose, 10 mM HEPES, 1.0 mM sodium pyruvate, 10% heat-inactivated FCS, 100 mg/mL Normocin™ (cat. no. ant-nr-1, Invivogen), 50 U/mL-50 µg/mL Pen-Strep, and with 200 µg/mL Zeocin™ (cat. no. ant-zn-1, Invivogen) at 37 °C with 5% CO_2_. Prior to experimentation, THP1-XBlue™ cells were differentiated into macrophage-like cells (dTHP1-XBlue cells) by incubation with 30 ng/mL phorbol 12-myristate-13-acetate (PMA, cat. no. P1585, Sigma-Aldrich) for 72 h, in the absence of Zeocin. As specified by the manufacturer, cells secrete Secreted Embryonic Alkaline Phosphatase (SEAP) upon NF-κB and AP-1 activation *via* TLR stimulation, which can be quantified using the QUANTI-Blue™ solution (cat. no. rep-qbs3, Invivogen).

##### HEK-Dual™ hTLR2 cells

Dual NF-κB/AP-1, IL-8 reporter human embryonic kidney 293 (HEK293) cells, stably co-transfected with the human toll-like receptor 2 (hTLR2) and CD14 genes, along with SEAP and Lucia luciferase reporter genes, and carrying a triple knockout of TLR3, TLR5 and TNFR (HEK-Dual™ hTLR2 cells, cat. no. hkd-htlr2ni, Invivogen), were cultured in DMEM (cat. no. D6546, Sigma-Aldrich) containing 4.5 g/L glucose, 2 mM L-glutamine, 10% heat-inactivated FCS, 100 µg/mL Normocin™, 100 U/mL-100 µg/mL Pen-Strep, 50 µg/mL of Zeocin™, and 100 µg/mL Hygromycin B Gold (cat. no. ant-hg-1, Invivogen) at 37 °C with 5% CO_2_. Upon NF-κB/AP-1 and/or IL-8 activation *via* TLR2 stimulation, HEK-Dual™ hTLR2 cells secrete SEAP and Lucia luciferase, which can be quantified using the QUANTI-Blue™ solution and the QUANTI-Luc™ (cat. no. rep-qlc1, Invivogen), respectively.

##### HEK-Blue™ TNF-α cells

NF-κB/AP-1 reporter HEK293 cells are stably co-transfected with the genes encoding for the human TNF-α receptor and a SEAP reporter gene under the control of the IFN-β minimal promoter fused to five AP-1 and five NF-κB binding sites (HEK-Blue™ TNF-α cells, cat. no. hkb-tnfdmyd, Invivogen), designed for the detection of bioactive human and murine tumor necrosis factor-alpha (TNF). Cells were cultured in DMEM containing 4.5 g/L glucose, 2 mM L-glutamine, 10% heat-inactivated FCS, 100 µg/mL Normocin™, 100 U/mL-100 µg/mL Pen-Strep, 100 µg/mL of Zeocin™, and 1 µg/mL of Puromycin (cat. no. ant-pr-1, Invivogen) at 37 °C with 5% CO2. Upon TNF stimulation, cells activate NF-κB/AP-1, leading to SEAP secretion. TNF-induced activation can be quantified using QUANTI-Blue™ solution.

##### HEK-Blue™ IL-1R cells

NF-κB/AP-1 reporter HEK293 cells, endogenously expressing the human IL-1 receptor and stably co-transfected with the murine IL-1 receptor and a SEAP reporter gene under the control of the IFN-β minimal promoter fused to five NF-κB and five AP-1 binding sites (HEK-Blue™ IL-1R cells, cat. no. hkb-il1r, Invivogen), designed to detect bioactive human and murine IL-1α and IL-1β, were cultured in DMEM containing 4.5 g/L glucose, 2 mM L-glutamine, 10% heat-inactivated FCS, 100 µg/mL Normocin™, 100 U/mL-100 µg/mL Pen-Strep, 100 µg/mL of Zeocin™, 1 µg/mL of Puromycin, and 200 µg/mL Hygromycin B Gold at 37 °C with 5% CO2. Following the binding of IL-1α and IL-1β to IL-1R1, cells activate NF-κB/AP-1, leading to SEAP secretion. IL-1α/β-induced activation can be quantified using QUANTI-Blue™ solution.

### Isolation and purification of *Ef*-EVs

Unless otherwise specified, *Ef*-EVs refer to EVs derived from *E. faecalis* type strain (DSM 20478, German Collection of Microorganisms and Cell Cultures (DSMZ)). *E. faecalis* DSM 20478 and *E. faecalis* clinical bloodstream isolates (clinical strain 1, 2, 3, and 4) were cultured in Brain Heart Infusion (BHI) medium (cat. no. 53286, Merck) or on BHI agar plates (BHI medium supplemented with 1.5% agar (cat. no. A1296, Sigma-Aldrich) under static conditions at 37 °C [38, 39]. A single colony of *E. faecalis* was inoculated into 10 mL of BHI medium and grown overnight at 37 °C. The overnight culture was then diluted into 400 mL of fresh BHI medium and incubated until reaching the late phase of exponential growth. Bacterial medium was harvested and centrifuged at 5000 × *g* for 15 min at 4 °C. The supernatant was carefully separated from the bacterial pellet by decantation and further filtered through 0.45 μm PVDF bottle-top filters (cat. no. 6–0039, Neolab) to remove any remaining bacteria. Absence of bacterial contamination was confirmed *via* overnight incubation of 0.5-1 mL of the filtrate on agar plates at 37 °C. The filtered supernatants were then loaded in 70 mL ultracentrifuge tubes (cat. no. 355655, Beckman Coulter) and ultracentrifuged (UC) at 160,000 × *g* for 3 h at 4 °C (rotor SW 45Ti, Optima L-90k, Beckman Coulter) to pellet the *Ef*-EVs. The supernatants were removed, and the EV pellets were resuspended in 100 µL of 0.2 μm-filtered PBS (cat. no. 99255, TPP, Switzerland). Size exclusion chromatography (SEC) was performed to separate EVs from proteins and diluents. An aliquot of 500 µL of EV pellet was loaded onto a 40 mL Sepharose CL-2B (cat. no 17-0140-01, GE Life Science) column (1.5 cm-diameter borosilicate glass Kimble^®^ Flex-Column^®^, cat. no. 420400-1530, DWK Life Sciences). Forty fractions of 1 mL were collected in 1.7 mL tubes (cat. no. MCT-175-A, Axygen, Corning Incorporated) by eluting with 0.2 μm filtered PBS. The protein concentration of the collected fractions was quantified using the bicinchoninic acid (BCA) assay (cat. no. QPBCA, Sigma Aldrich) according to the manufacturer’s instructions. The total protein concentration in each fraction was determined by interpolation from a standard calibration curve generated using bovine serum albumin (BSA). The fraction with the highest EV content was selected for further physicochemical and biological analyses, as previously described [43, 44]. Fractions were stored at -80 °C until further use.

#### Fluorescent labeling of *Ef*-EVs

Pelleted *E. faecalis* DSM 20478-derived EVs obtained after UC were fluorescently labeled with DiI (cat. no. V22885, Vybrant DiI Cell Labeling Solution, Thermo Fisher), using 2 µL of dye per mL of EV pellet suspension (final DiI concentration: 2 µM), followed by incubation for 30 min at 37 °C. Unincorporated dye and impurities were separated from DiI-labeled *Ef*-EVs by SEC, as previously described. Fractions with the highest fluorescence intensity and EV content were selected for subsequent analysis [43].

### Formulation and functionalization of small unilamellar vesicles

Small unilamellar vesicles (SUVs) were prepared by thin film hydration followed by extrusion, using the following lipid composition: 79 mol% Egg L-α-phosphatidylcholine (Egg PC, cat. no. 840051, Avanti Polar Lipids), 20 mol% Egg L-α-phosphatidylglycerol (Egg PG, cat. no. 841138, Avanti Polar Lipids), and 1 mol% 1,2-dioleoyl-sn-glycero-3-phosphoethanolamine-N-(Cyanine 5) (18:1 Cyanine 5 PE, cat. no. 810335, Avanti Polar Lipids) [45]. Briefly, lipids dissolved in chloroform were mixed at given ratios in glass vials and dried under vacuum for 40 min at room temperature (RT) and dark conditions. The formed lipid film was rehydrated with PBS at a total lipid concentration of 6 mM and incubated for 20 min at RT, protected from light. SUV suspensions were obtained after vortexing and extrusion of the resulting liposome suspension through a 100 nm pore size polycarbonate membrane (cat. no. 610005, Avanti Polar Lipids, USA) at least 13 times. SUVs were then functionalized with synthetic bacterial lipopeptide Pam_3_-Cys-Ser-Lys_4_ (Pam_3_CSK_4_, cat. no. tlrl-pms, Invivogen) by post-insertion. In brief, SUVs at 120 µM were incubated with Pam_3_CSK_4_ at various concentrations (0.4, 0.04, or 0.004 mol% of total SUV lipid composition) in PBS at 37 °C for 15 min and dark conditions, resulting in Pam_3_CSK_4_-SUVs. SUV and Pam_3_CSK_4_-SUV suspensions were stored at 4 °C.

### Particle characterization

#### Nanoparticle tracking analysis

Particle size distribution and concentration of *Ef*-EV, SUV, and Pam_3_CSK_4_-SUV suspensions were characterized by nanoparticle tracking analysis (NTA). Samples were diluted in 0.2 μm filtered PBS to achieve 20–120 particles per frame before measurements. All samples, except EVs from bloodstream-derived *E. faecalis* clinical strains, were analyzed using an LM-10 instrument (Malvern) equipped with a 532-nm laser, recording three 30-second high-sensitivity videos (camera level 13–15). Video acquisitions were processed using the NanoSight software version no. 3.4 build 3.4.4 (Malvern). For the size comparison study of EVs derived from *E. faecalis* clinical bloodstream isolates and the type strain *E. faecalis* DSM 20478, EV samples were analyzed using an LM-10 instrument equipped with a 405-nm laser, recording at least three 30-second high-sensitivity videos (camera level 14) that were processed by the NanoSight software version no. 2.3 build 0017 (Malvern).

#### Cryo-TEM imaging

Purified EVs were subjected to cryogenic transmission electron microscopy (cryo-TEM). To this end, a 2 µL sample was placed onto a holey carbon grid (type S147-4, Plano, Wetzlar) and blotted for 2 s before being rapidly submerged into liquid ethane at a temperature of -165 °C using a Gatan (Pleasanton) CP3 cryo plunger. The sample was then transferred under liquid nitrogen to a Gatan model 914 cryo-TEM sample holder. Low-dose TEM bright-field imaging was conducted at a temperature of -173 °C using a JEM-2100 LaB_6_ microscope (JEOL) operating at an accelerating voltage of 200 kV. Images were acquired at a resolution of 1024 × 1024 pixels using a Gatan Orius SC1000 CCD camera with an imaging time of 4 s and a binning factor of 2.

### NF-κB/AP-1 reporter assay

NF-κB/AP-1 reporter cells were submitted to 24-hour treatments at 37 °C with 5% CO_2_. Afterward, their NF-κB/AP-1 activity was quantified by the QUANTI-Blue™ solution assay, according to the manufacturer’s instructions. For all experiments, cells treated with growth medium alone served as negative controls. To evaluate if *Ef*-EVs derived from *E. faecalis* DSM 20478 induce NF-κB/AP-1 activation, dTHP1-XBlue cells (1 × 10^5^ cells per well in a 96-well plate) were treated with 200 µL/well of fresh medium containing *Ef*-EVs at various concentrations (100; 500; 1000; 5000; 10,000; 50,000 EVs/cell). LPS (100 ng/mL) was used as a positive control. To compare the inflammatory effect induced by *Ef*-EVs derived from *E. faecalis* clinical bloodstream isolates with those induced by *Ef*-EVs derived from *E. faecalis* DSM 20478, dTHP1-XBlue cells (1 × 10^5^ cells per well in a 96-well plate) were treated with 200 µL/well of fresh medium containing *Ef*-EVs at increasing concentrations (1000, 5000, and 10,000 EVs/cell). LPS (100 ng/mL) and Pam_3_CSK_4_ (100 ng/mL) were used as positive controls. To elucidate whether *Ef*-EVs induce NF-κB/AP-1 activation by TLR2 engagement, dTHP1-XBlue were pre-incubated for 1 h with fresh medium containing 1 µg/mL of neutralizing monoclonal antibodies (anti-hTLR2-IgA mAb, cat. no. maba2-htlr2-2, Invivogen) or human IgA2 control (cat. no. maba2-ctrl, Invivogen) before adding either *Ef*-EVs (7000 EVs/cell), ultrapure LPS from *E. coli* K12 (LPS, 1 ng/mL, cat.no. tlrl-peklps, Invivogen), or Pam_3_CSK_4_ (1 ng/mL) in antibody-containing medium. Cell treatments consisting of LPS at 1 ng/mL, Pam_3_CSK_4_ at 1 ng/mL, or *Ef*-EVs at 7000 EVs/cell were used as positive controls. Treatments did not impair cell viability (data not shown). Post-treatment, 20 µL of supernatant was collected from each well, mixed with 180 µL of QUANTI-Blue™ solution, and incubated at 37 °C for at least 1 h. The product of the colorimetric reaction triggered by the SEAP present in the collected supernatants was measured with a microplate reader (GloMax^®^ Discover Multimode Microplate Reader, Promega) at 600 nm [46].

### Cytokine detection assay

The amounts of TNF as well as IL-1α and IL-1β secreted by dTHP1-XBlue cells following treatment with *Ef*-EVs derived from *E. faecalis* DSM 20478 or bloodstream-derived *E. faecalis* clinical strains were quantified as follows. First, dTHP1-XBlue cells (1 × 10^5^ cells per well in a 96-well plate) were treated with 200 µL/well of fresh medium containing *Ef*-EVs at increasing concentrations (1000, 5000, and 10,000 EVs/cell) and incubated at 37 °C with 5% CO_2_. LPS (100 ng/mL) and Pam_3_CSK_4_ (100 ng/mL) were used as positive controls. After 4 h of treatment, the concentration of TNF as well as the combined concentration of IL-1α and IL-1β in the cell culture supernatants were quantified using HEK-Blue™ TNF-α and HEK-Blue™ IL-1R cells, respectively, according to the manufacturer’s instructions. Supernatants were diluted 10-fold in test medium (DMEM containing 4.5 g/L glucose, 2 mM L-glutamine, 10% heat-inactivated FCS, 100 µg/mL) prior to cytokine detection. Then, 20 µL/well of sample was added to a 96-well plate, followed by 180 µL/well of either HEK-Blue™ TNF-α or HEK-Blue™ IL-1R cell suspension (0.5 × 10^5^ cells per well). Plates were incubated overnight at 37 °C with 5% CO_2_. After incubation, 20 µL of HEK-Blue™ TNF-α and HEK-Blue™ IL-1R cell supernatant from each well was collected, mixed with 180 µL of QUANTI-Blue™ solution, and incubated at 37 °C for 2 h. SEAP levels were then measured as described above. Cytokine concentrations were determined by interpolation from a standard curve, generated with recombinant human TNF (1 pg/mL − 10 ng/mL, cat. no. rcyc-htnfa, Invivogen) or recombinant human IL-1β (0.01 pg/mL − 10 ng/mL, cat. no. rcyc-hil1b, Invivogen).

### Assessment of EV uptake as prerequisite for immune activation

#### Evaluation of TLR2-mediated uptake of *Ef*-EVs

HEK-Dual™ hTLR2 and dTHP1-XBlue cells were seeded in 12-well plates at 5 × 10^5^ cells per well in 1 mL of medium. Cells were pretreated for 1 h at 37 °C with 5% CO_2_ in growth medium containing either anti-hTLR2-IgA mAb (1 µg/mL) or human IgA2 control mAb (1 µg/mL). Cells were then treated with DiI-labeled *Ef*-EVs (7000 EVs/cell) while maintaining antibody concentrations. After 4 and 24 h of incubation, dTHP1-XBlue cells were washed with PBS and detached using Accutase (cat. no. A6964, Sigma-Aldrich). Following the manufacturer’s instructions, HEK-Dual™ hTLR2 cells were washed and detached in PBS. As previously described, cells were then centrifuged at 500 × *g* for 4 min, re-suspended in PBS containing 2% FCS, and cellular uptake of EVs was immediately measured *via* flow cytometry (LSRFortessa, BD Bioscience). EV fluorescence was acquired in the phycoerythrin (PE) channel using the 561-nm laser for excitation and a 582/15 bandpass filter for emission detection. Furthermore, the NF-κB/AP-1 activity of the cells was measured after 24 h of incubation, as previously described.

EV uptake into dTHP1-XBlue cells was also assessed by confocal laser scanning microscopy (LSM710, Carl Zeiss AG) after 24 h of incubation. Briefly, cells (0.75 × 10^5^ cells per well) were plated and differentiated in µ-Slide 8-well chambered coverslip (cat. no. 80826, ibidi) in 300 µL medium per well. Cells were pretreated for 1 h at 37 °C with 5% CO_2_ in growth medium containing either anti-hTLR2-IgA mAb (1 µg/mL) or human IgA2 control mAb (1 µg/mL), followed by treatment with DiI-labeled *Ef*-EVs (7000 EVs/cell) while maintaining antibody concentrations. After 24 h of incubation, cells were washed with PBS to remove non-internalized EVs. Afterward, the membrane of cells was labeled by incubation with 5 µg/mL wheat germ agglutinin (WGA) fluorescein conjugate (cat. no. W834, Thermo Fisher) for 10 min at 37 °C. Cells were then fixed with 4% paraformaldehyde (PFA) in PBS for 15 min at RT. Nuclei were stained by incubating the fixed cells with 1 µg/mL of 4′,6-Diamidino-2-phenylindole dihydrochloride (DAPI, cat. no. 32670, Sigma-Aldrich) for 30 min at RT. Fluorescent spectra from DiI, fluorescein, and DAPI were recorded using a lambda scan with a C-Apochromat 63x/1.2 W Corr M27 objective lens (cat. no. 421787-9970-799, Carl Zeiss AG). Micrographs were extracted from lambda scans through linear unmixing using ZEN Black Edition 2012 (version no. 8.1, Carl Zeiss AG).

To further elucidate TLR2 engagement in EV uptake, internalization of Pam_3_CSK_4_-SUVs was monitored in dTHP1-XBlue cells. In detail, cells (5 × 10^5^ cells per well in 1 mL of medium in 12-well plates) were treated with 6 µM of SUVs composed of different amounts of Pam_3_CSK_4_ (0, 0.4, 0.04, or 0.004 mol% of total SUV lipid composition) for 18 h at 37 °C with 5% CO_2_. Alternatively, similar to previous experimental setups, cells were pretreated for 1 h at 37 °C with 5% CO_2_ in growth medium containing either anti-hTLR2-IgA mAb (1 µg/mL) or human IgA2 control mAb (1 µg/mL). Cells were then treated for 18 h with Pam_3_CSK_4_-SUVs (0.04 mol% of the total SUV lipid composition) at 37 °C with 5% CO_2_, while maintaining the antibody concentrations. Following treatment, SUV uptake was measured *via* flow cytometry. SUV fluorescence (Cy5) was detected in the allophycocyanin (APC) channel using the 640-nm laser for excitation and a 660/20 bandpass filter for emission detection. NF-κB/AP-1 activity of cells was quantified after 18 h of incubation, as previously described.

#### Toxicity screening of pharmacological inhibitors of endocytosis

The cytotoxic effects of pharmacological inhibitors of endocytosis were assessed in dTHP1-XBlue cells, which were plated and differentiated at a density of 1 × 10^5^ cells per well in 96-well plates with 200 µL of medium per well. Cells were exposed to varying concentrations of dynamin Inhibitor I (dynasore, from 20 µM to 200 µM, cat. no. 324410, Sigma-Aldrich), chloroquine diphosphate salt (chloroquine, from 10 µM to 100 µM, cat. no. C6628, Sigma-Aldrich), chlorpromazine hydrochloride (chlorpromazine, from 10 µM to 100 µM, cat. no. C8138, Sigma-Aldrich), amiloride hydrochloride hydrate (amiloride, from 20 µM to 200 µM, cat. no. A7410, Sigma-Aldrich), InSolution™ LY 294,002 (LY 294002, from 10 µM to 100 µM, cat. no. 440204, Sigma-Aldrich), and Cytochalasin D (CytD, from 0.1 µM to 40 µM, cat. no. C8273, Sigma-Aldrich) in 200 µL of fresh medium. After 4.5 h of incubation, the supernatants were aspirated, and cells were treated with 150 µL of 3-(4,5-dimethylthiazol-2-yl)-2,5-diphenyltetrazolium bromide (MTT) solution (0.5 mg/mL in medium, cat. no. M5655, Sigma Aldrich) for 1 h at 37 °C with 5% CO_2_. Subsequently, the supernatant was removed, and formazan crystals were dissolved by adding 100 µL of DMSO per well. Absorbance was measured at 560 nm using a microplate reader (GloMax^®^ Discover Multimode Microplate Reader).

#### Pharmacological inhibition of cellular uptake of *Ef*-EVs

The routes of *Ef*-EV uptake were studied using pharmacological inhibitors of endocytosis. Briefly, dTHP1-XBlue cells were plated and differentiated in 12-well plates at a seeding density of 5 × 10^5^ cells in 1 mL of medium per well. Cells were pretreated for 30 min at 37 °C with 5% CO_2_ in growth medium containing one of the following inhibitors: 100 µM of dynasore, 100 µM of chloroquine, 20 µM of chlorpromazine, 120 µM of amiloride, 100 µM of LY 294,002, or 5 µM and 40 µM of CytD. DiI-labeled *Ef*-EVs (7000 EVs/cell) were then added while maintaining the inhibitor concentrations. After 4 h of EV treatment, EV uptake was measured by flow cytometry as described above. EV fluorescence was acquired in the PE channel.

To evaluate potential fluorescence artifacts produced by unincorporated DiI, a control EV-mock solution (DiI control) was prepared by subjecting DiI resuspended in PBS (final DiI concentration: 2 µM) to SEC under identical conditions used for DiI-labeled *Ef*-EVs. The fraction corresponding to the EV elution profile was collected and applied to dTHP1-XBlue cells following the same treatment protocol used for labeled EVs. Potential nonspecific DiI-associated signals were assessed by acquiring dye fluorescence in the PE channel.

#### Assessment of immune activation during EV uptake inhibition

To investigate whether EV uptake is required to initiate inflammatory signaling, EV-induced NF-κB/AP-1 activity was assessed during inhibition of *Ef*-EV endocytosis. dTHP1-XBlue cells (1 × 10^5^ cells per well in a 96-well plate with 200 µL of medium per well) were pretreated for 30 min at 37 °C with 5% CO_2_ in growth medium containing 100 µM of dynasore. *Ef*-EVs (7000 EVs/cell) were then added while maintaining the inhibitor concentrations. Cell treatment consisting of *Ef*-EVs at 7000 EVs/cell was used as a positive control. Cells treated with growth medium alone and growth medium containing 100 µM of dynasore served as negative controls. After 24 h of treatment, NF-κB/AP-1 activity was quantified as described above.

### Cell viability assay

HUVECs and HMDMs were seeded in 96-well plates at a density of 1 × 10^4^ and 4 × 10^4^ cells per well, respectively. Both cell types were exposed to varying concentrations of *Ef*-EVs (1000, 5000, and 10,000 EVs per cell) in 200 µL of fresh medium. After 24 h of incubation, the supernatants were aspirated, and cells were treated with 150 µL of MTT solution (0.5 mg/mL in medium) for 2 h at 37 °C with 5% CO_2_. Subsequently, the supernatant was removed, and formazan crystals were dissolved by adding 100 µL of DMSO per well. Absorbance was measured at 560 nm using a microplate reader (GloMax^®^ Discover Multimode Microplate Reader).

### Macrophage morphology analysis

HMDMs were cultured and treated with *Ef*-EVs as described above. Changes in cell morphology were monitored using the IncuCyte S3 (Sartorius) equipped with the Cell-by-Cell Analysis Software Module (cat. no. 9600-0031, Sartorius). Cells were grouped based on their eccentricity into either round or elongated shapes [42].

### Gene expression in HMDMs and HUVECs

HMDMs (2.5 × 10^5^ cells per well in a 24-well plate) and HUVECs (1 × 10^5^ cells per well in a 24-well plate) were treated with *Ef*-EVs for 24–48 h (1000, 5000, and 10,000 EVs/cell in 500 µL medium). Three individual donors were used for each cell type. Total RNA was isolated using the Direct-zolTM RNA MiniPrep Kit (cat. no. R2052, Zymo Research). The concentration of isolated RNA was quantified by NanoDrop™ (Thermo Fisher Scientific). Equal amounts of RNA were reverse transcribed using the High Capacity cDNA Reverse Transcription Kit (cat. no. 4368813, Thermo Fisher Scientific) in the presence of RNase inhibitor (cat. no.10777-019, Invitrogen) according to the manufacturer’s instructions. Amplifications were carried out in 10 µL reaction solutions containing 0.25 µL of each primer (10 µM), and 2 µL of 5x Hot FIREPol EvaGreen qPCR Mix (cat. no. 08-24-00020, Solis BioDyne). The primer sequences for each transcript are detailed in Table 1. PCR assays were performed in a CFX96 touch™Real-Time PCR detection system (BioRad). Data were normalized to the beta-actin housekeeping gene (*ACTB*).

**Table 1:** Primer sequences used for qPCR

Gene	Accession number	Primer forward sequence	Primer reverse sequence
*ACTB*	NM_001101.3	TGCGTGACATTAAGGAGAAG	GTCAGGCAGCTCGTAGCTCT
*CCL2*	NM_002982.4	TTGATGTTTTAAGTTTATCTTTCATGG	CAGGGGTAGAACTGTGGTTCA
*CXCL8*	NM_000584.4	GAGAAGTTTTTGAAGAGGGCTGA	GCTTGAAGTTTCACTGGCATCT
*ICAM*	NM_000201.3	TGACCGTGAATGTGCTCTCC	TCCCTTTTTGGGCCTGTTGT
*IL10*	NM_000572	CAACAGAAGCTTCCATTCCA	AGCAGTTAGGAAGCCCCAAG
*IL1A*	NM_000575.5	GCGTTTGAGTCAGCAAAGAAGT	CATGGAGTGGGCCATAGCTT
*IL1B*	NM_000576.3	GGCTGCTCTGGGATTCTCTT	AGTCATCCTCATTGCCACTGTAA
*IL6*	NM_000600.5	ACATCCTCGACGGCATCTCA	TCACCAGGCAAGTCTCCTCATT
*NOS3*	NM_001160109.1	AACCCCAAGACCTACGTGC	CATGGTAACATCGCCGCAGA
*TLR2*	NM_003264.3	GGAGTTCTCCCAGTGTTTGGT	GCAGTGAAAGAGCAATGGGC
*TNF*	NM_000594.4	CTCCACCCATGTGCTCCTCA	CTCTGGCAGGGGCTCTTGAT
*TSC22D3*	NM_004089.3	CATGTGGTTTCCGTTAAGCTGG	AGGATCTCCACCTCCTCTCTC
*VCAM*	NM_001078.4	TTTGGATAATGTTTGCAGCTTCTCA	CACCTTCCCATTCAGTGGACTA
*VEGFA*	NM_001171623.1	CGCTTACTCTCACCTGCTTCTG	GGTCAACCACTCACACACACAC

### Uptake of *Ef*-EVs in HMDMs and HUVECs

HMDMs (2.5 × 10^5^ cells per well) were seeded in 24 well plates with 500 µL of medium per well, while HUVECs (1 × 10^5^ cells per well) were seeded in 12-well plates with 1 mL of medium per well. Cells were treated with DiI-labeled *Ef*-EVs (30,000 EV/cell) and incubated at 37 °C with 5% CO_2_ for up to 48 h. After 24 and 48 h of incubation, cells were washed with PBS and detached using Accutase. Cells were then centrifuged at 500 × *g* for 4 min, re-suspended in PBS containing 2% FCS, and cellular uptake of EVs was immediately measured *via* flow cytometry. EV fluorescence was acquired in the PE channel.

### Flow cytometry data collection and analysis

For all flow cytometry experiments, data were recorded using BD FACSDiva software (version no. 8.0.1, BD Bioscience) and further analyzed in FlowJo software (version no. 10.10.0, BD Bioscience). Analyses were exclusive to the evaluation of fluorescence intensity associated with singlet cells after the discrimination of cell debris and clumps.

### Extracellular flux analysis

Extracellular acidification rate (ECAR) was assessed using the Seahorse XFe-96 Flux Analyzer (Agilent, USA) with the Glycolysis Stress Test Kit (cat. no. 103020–100, Agilent), following the manufacturer’s recommendations. Briefly, HMDMs were seeded at a density of 8 × 10^4^ cells per well in XF-96-cell culture plates (cat. no. 103793–100, Agilent) and stimulated for 24 h with Pam_3_CSK_4_ (10 ng/mL), *Ef*-EVs (10,000 and 50,000 EVs/cell), or left untreated. Before the assay, cells were washed, and the medium was replaced with Seahorse XF RPMI medium, pH 7.4 (cat. no. 103681-100, Agilent) without glucose, phenol red, and sodium bicarbonate, supplemented with 5 mM HEPES and 2 mM L-glutamine. Cells were incubated at 37 °C for 1 h before the assay. The assay run was carried out in standard conditions (i.e., 3 injection cycles with a 3-minute mixing phase and a 3-minute measurement phase) using Glucose (final concentration: 25 mM), Oligomycin (final concentration: 1.5 µM), and 2-Deoxyglucose (final concentration: 50 mM) [47]. Hoechst 33,342 (cat. no. H3570, Thermo Fisher) was added at the final concentration of 20 µM for nuclear staining. Following the assay, the cell number per well was quantified by nuclei counting using Hoechst fluorescence detection on a Tecan Spark Cyto (Tecan), and analyzed with Image Analyzer™ software (Tecan). ECAR values were then normalized to Hoechst-positive nuclei counts and analyzed using Wave software (version 2.6.4, Agilent).

### In vivo zebrafish embryo model

#### Zebrafish husbandry

Zebrafish husbandry and all experimental procedures were conducted in compliance with the European Union Directive 2010/63/EU on the protection of animals used for scientific purposes and the German Animal Welfare Act (§ 11 Abs. 1 TierSchG). Zebrafish were maintained according to standard protocols [48] in an automated aquatic housing system (PENTAIR, Apopka) with regular monitoring to ensure the following conditions: pH 7.0 ± 0.1, temperature 28 ± 0.5 °C, conductivity 800 ± 50 µS, and a light-dark cycle of 14 h–10 h. The transgenic zebrafish line Tg(mpeg1.1:GFP)ka101, with green-fluorescent embryonic macrophages, was used in this study. Embryos and larvae were raised in fresh 0.3 × Danieau’s medium (17 mM NaCl, 2 mM KCl, 0.12 mM MgSO_4_, 1.8 mM Ca(NO_3_)_2_, 1.5 mM HEPES, 1.2 µM methylene blue, pH 7.1) at 28 °C. Larvae were euthanized by submersion in ice water for at least 12 h, no later than 120 h post-fertilization.

#### Maximum tolerated concentration

The maximum tolerated concentration (MTC) test was conducted as previously reported [49]. Zebrafish larvae (*n* = 20) were injected with 4 nL of either *Ef*-EVs (200,000 EVs) or PBS at the 3rd-day post fertilization (dpf) into the yolk sac.

#### Injection of *Ef*-EVs into the yolk sac and sample preparation

The injection and sample preparation for macrophage isolation were performed as previously described [49]. Zebrafish larvae were injected either with 4 nL of *Ef*-EVs or PBS (*N* = 3, 100–170 larvae/condition, 200,000 EVs/larva) into the yolk sac. Larvae were anesthetized in 250 µg/mL tricaine (ethyl 3-aminobenzoate methanesulfonate, cat. no. E10521, Sigma-Aldrich) 18 h after injection. Larvae were then homogenized using a 70 μm cell strainer and a syringe plunger. Cells were washed with cold buffer (PBS, 2 mM EDTA, 2% FCS), and centrifuged at 400 × *g* for 5 min at 4 °C. Pellets were re-suspended in the buffer, filtered through a 40 μm cell strainer, and centrifuged at 400 × *g* for 5 min at 4 °C. Cells were re-suspended in buffer and kept on ice until FACS analysis.

#### Fluorescence‑activated cell sorting (FACS) analysis

Forward scatter (FSC) and side scatter (SSC) were employed to identify cells while excluding cell debris. Initial tests confirmed the absence of doublet formation, enabling direct fluorescence gating. Cells with autofluorescence were excluded by gating parameters established by the use of wild-type zebrafish larvae (AB line, lacking GFP+ macrophages). Cells were then sorted based on their endogenous eGFP expression in the FL2 channel (λ_ex_ = 488 nm /λ_em_ = 525 nm) using the SH800S cell sorter (Sony).

#### RNA library preparation

Libraries were prepared from 20 ng of total RNA. For mRNA sequencing, a modified SmartSeq 2 protocol was applied. Briefly, 20 ng of total RNA per sample were used as input. RNA was primed by adding Oligo-dT Primer (5´AAGCAGTGGTATCAACGCAGAGTACTTTTT TTTTTTTTTTTTTTTTTTTTTTTTTVN, where V = A/C/G and N = any base, final concentration: 1 × 10^− 6^ M, 1 × 10^− 3^ M dNTPs (final concentration)) followed by a denaturation step at 72 °C for 3 min and immediate cooling on ice.

Reverse transcription was performed in a 10 µL volume reaction by using 0.5 µL Superscript II RT (200 U/µL, cat. no. 18064022, Thermo Fisher Scientific), 0.4 µL RNAse inhibitor (40 U/µL, cat. no. N2515, Promega), 5 × 10^− 3^ M dithiothreitol (DTT), 1 M Betaine, 6 × 10^− 3^ M MgCl_2_, and 1 × 10^− 6^ M TSO (B-AAGCAGTGGTATCAACGCAGAGTACAT997, B = 5’ biotin, 7 = LNA g, 9 = RNA‐G) under the following incubation conditions: 42 °C for 90 min, 10 × cycling of 50 °C for 2 min and 42 °C for 2 min, finalized by 70 °C for 15 min.

The preamplification of the cDNA was carried out using the KAPA HiFi HotStar Ready Mix (cat. no. KK2601, Roche) and 0.1 × 10^− 6^ M of the IS PCR primers (5´AAGCAGTGGTATCAACGCAGAGT) in a 25 µL volume reaction under the following PCR conditions: 98 °C for 3 min, 15 × cycling of 98 °C for 20 s, 67 °C for 15 s, 72 °C for 6 min and a final elongation at 72 °C for 5 min. The cDNA was purified using 0.8 × Agencourt AMPure XP Beads (cat. no. A63881, Beckman Coulter) and quantified with the help of the Qubit dsDNA HS Assay Kit (cat. no. Q32851, Thermo Fisher Scientific). cDNA integrity was examined *via* the analysis of the fragment size distribution by using a Qsep1 (Bioptic).

The libraries were prepared by applying a tagmentation-based approach using the Nextera DNA Library Preparation Kit (Illumina, FC‐131‐1024). For each cDNA, 8 ng were tagmented for 10 min at 55 °C using 1 µL of the Tagment DNA Enzyme 1 in 20 µL reaction, immediately followed by the purification of the tagmented fragments using the MinElute PCR Purification Kit (cat. no. 28004, Qiagen) according to the manufacturer’s instructions. The amplification of the libraries was performed in a 30 µL reaction using the NEBNext High‐Fidelity 2X PCR Master Mix (cat. no. M0541S, New England Biolabs) and 0.33 × 10^− 6^ M indexed adapters (5′AATGATACGGCGACCACCGAGATCTACAC[i5]TCGTCGGCAGCGTC and 5″CAAGCAGAAGACGGCATACGAGAT[i7]GTCTCGTGGGCTCGG; Illumina). The PCR conditions were: 75 °C 5 min, 98 °C 30 s, 9 × cycling of 98 °C 10 s, 63 °C 30 s, and 72 °C 1 min, finalized by a long elongation at 72 °C for 5 min. The libraries were purified using 0.9 × Agencourt AMPure XP Beads, and the final concentration was determined with qPCR using the NEBNext Library Quant Kit for Illumina for Illumina (New England Biolabs).

#### mRNA sequencing data processing and analysis

The libraries were sequenced on the Aviti platform (Element Biosciences) in 2 × 75 nt mode for 15–30 million reads per sample. Raw reads were processed using the nf-core/rnaseq pipeline (version no. 3.13.0dev, nf-core) for mRNA seq with Nextflow software (version no. 23.04.1, Seqera Labs). Briefly, FASTQ reads were adapted and quality-trimmed with Trim Galore! (version no. 0.4.2, Babraham Bioinformatics), and the reads were aligned to the GRCz10 reference genome using Grape-NF (version no. 433e7621f6), which combines STAR (version no. 2.4.0j) for the alignment and RSEM (no. version 1.2.21) for the read assignment. Differential gene expression (DGE) analysis was carried out in R (version no. 4.4.3, The R Foundation) with the DESeq2 package. DGE was considered by a log2 fold change of 1 (fold change of 2) and a p-value < 0.05. Principal component analysis was performed using the transformed transcripts per kilobase million (TPM) values of all annotated protein-coding genes. Volcano plot and heatmap show all differentially expressed genes (DEGs) (p-value < 0.05). Subsequently, the TPM values of the DEGs were subjected to k-means clustering with iDEP.96 [50]. The raw and processed data were stored in the Gene Expression Omnibus (GEO) database under the accession number GSE304960.

### Statistics

If not stated differently, results are presented as mean ± standard deviation (SD), where N indicates the number of independent experiments and n the number of replicates per experiment. Statistical analyses, specified in the figure legends, were conducted using data from at least three independent experiments using GraphPad Prism software (version no. 10.4.1, GraphPad). Statistical significance was established at *p* < 0.05.

## Results

### Isolation and characterization of *Ef*-EVs

**Fig. 1:**
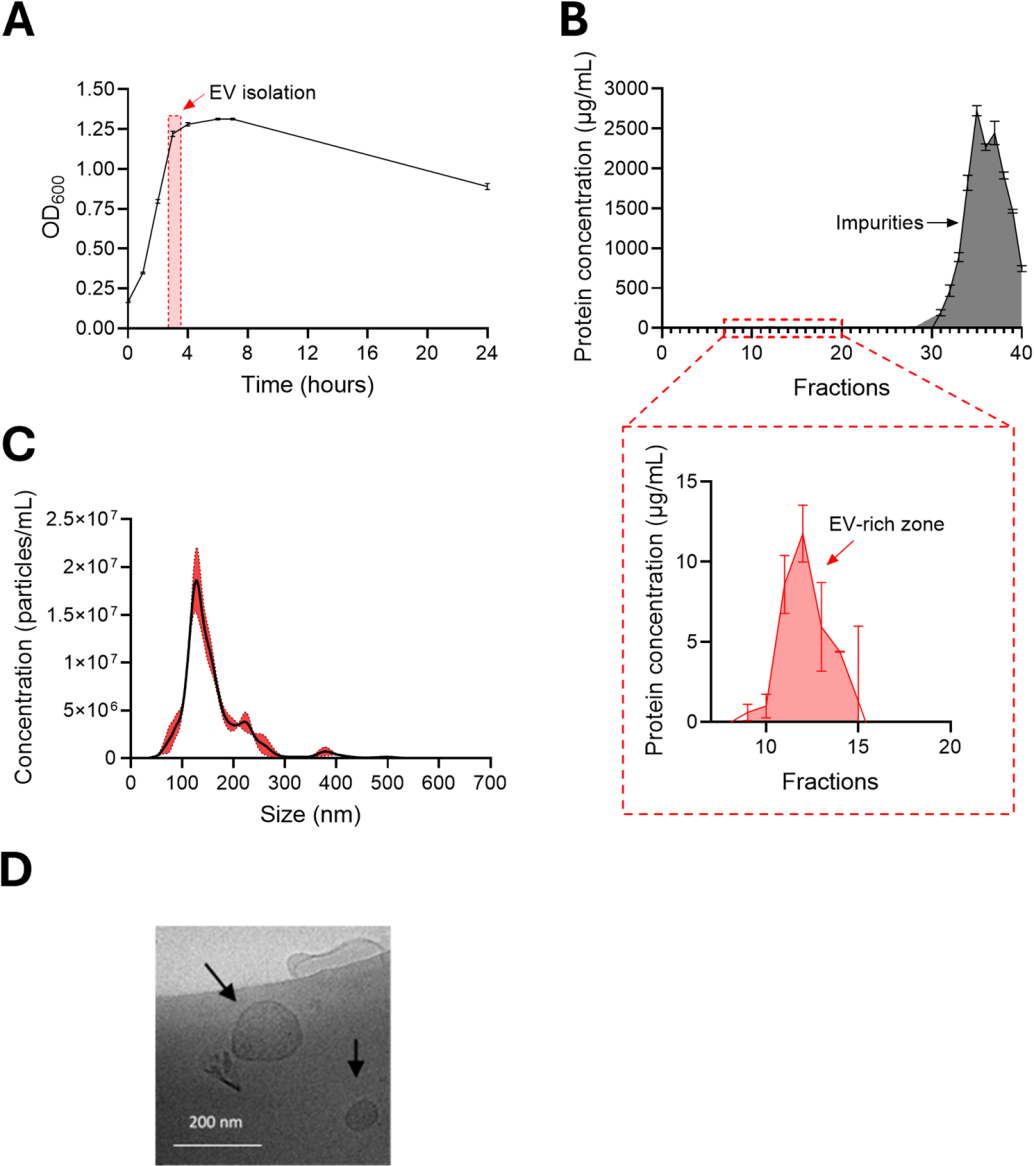
Isolation and characterization of *Ef*-EVs. **A** Growth curve of *E. faecalis* DSM 20478. The optical density (OD_600_) was measured from *E. faecalis* cultures in BHI medium grown under static conditions at 37 °C. Results are shown as mean ± SD (*N* = 3, *n* = 3). **B** Representative chromatogram obtained by protein concentration analysis of eluted fractions after size exclusion chromatography. Protein concentration was quantified by the BCA assay. The upper panel indicates the protein concentration of the first forty 1-mL fractions, indicating the elution peak corresponding to impurities (black area). The zoomed lower panel shows the protein concentration of the first 20 fractions, highlighting the EV-rich zone (red area). Results are shown as mean ± SD (*n* = 3). **C** Representative size distribution of particles in the vesicle-richest fraction by Nanoparticle Tracking Analysis. **D** Representative cryo-TEM image of *Ef*-EVs in the vesicle-richest fraction (scale bar = 200 nm)

EVs were isolated from *E. faecalis* DSM 20478 cultures at the late phase of exponential growth (Fig. 1A). After EV concentration by UC, *Ef*-EVs were purified by SEC, collecting 40 fractions of 1 mL each. The protein content in the eluted fractions was quantified using a BCA assay (Fig. 1B). Particle size distribution and particle concentration of *Ef*-EV suspensions were characterized by NTA (Fig. 1C), indicating an average particle mean size of 167.7 ± 13.6 nm, with a mode size of 134.6 ± 6.6 nm, and concentration of 1.067 × 10^11^ ± 0.28 × 10^11^ particles per milliliter. Morphology characterization of *Ef*-EVs by cryo-TEM confirmed their spherical, membrane-bound structure (Fig. 1D).

### Size comparison of *Ef*-EVs derived from clinical *E. faecalis* bloodstream isolates

**Fig. 2:**
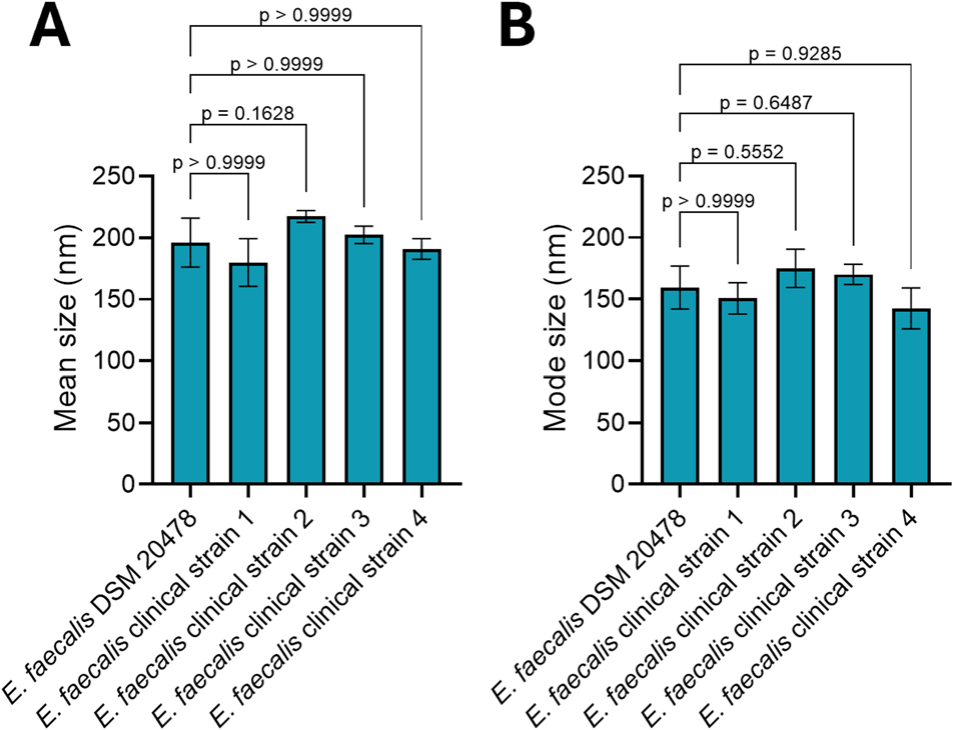
Particle size characterization of EVs derived from clinical *E. faecalis* bloodstream isolates and the type strain *E. faecalis* DSM 20478 as measured by NTA. **A** Average mean size and (**B**) average mode size of *Ef*-EVs. Results are presented as mean ± SD (*N* = 1, *n* ≥ 3) and were analyzed by Kruskal-Wallis test followed by Dunn’s multiple comparison *post hoc* test

EVs were isolated from cultures of four different clinical *E. faecalis* bloodstream isolates and the type strain *E. faecalis* DSM 20478 at the late phase of exponential growth (Figure S1). EVs were purified and characterized by NTA (Figure S2). The resulting mean size and mode size of EVs ranged from ~ 180 to 217 nm and ~ 143 to 175 nm, respectively (Fig. 2). No statistically significant differences in size were observed among *Ef*-EVs derived from the analyzed strains.

### *Ef*-EVs induce NF-κB/AP-1 activation in human reporter macrophages *via* TLR2 signaling

**Fig. 3:**
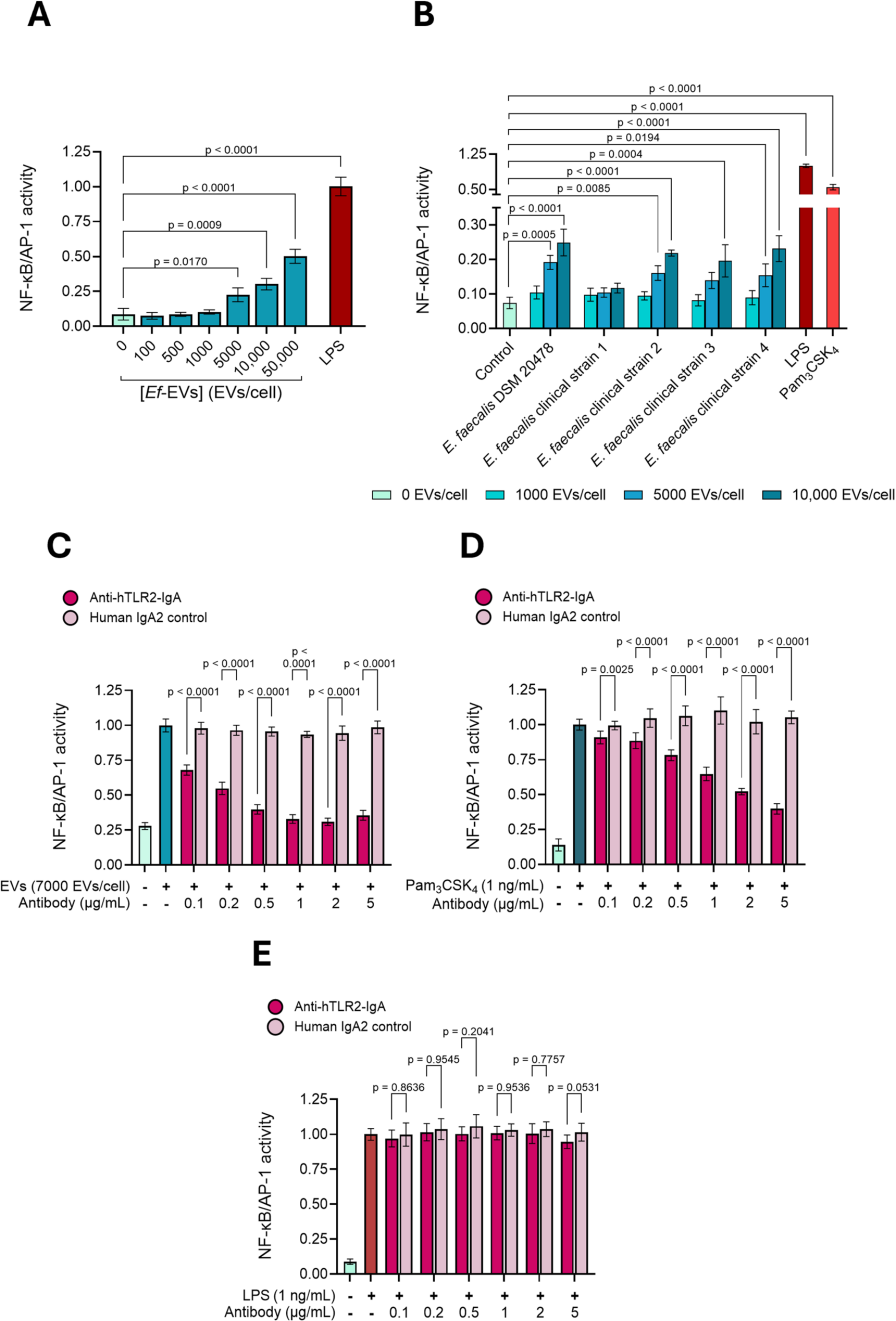
*Ef*-EVs activate the NF-κB/AP-1 pathways in human reporter macrophages *via* TLR2 signaling. **A** dTHP1-XBlue cells were treated with *Ef*-EVs (100 − 50,000 EVs/cell). LPS (100 ng/mL) was used as a positive control. **B** dTHP1-XBlue cells were treated with EVs derived from clinical *E. faecalis* bloodstream isolates (1000-10,000 EVs/cell). LPS (100 ng/mL) and Pam_3_CSK_4_ (100 ng/mL) were used as positive controls. C-E) dTHP1-XBlue cells were pretreated with increasing concentrations (from 0.1 to 5 µg/mL) of anti-hTLR2-IgA mAb (dark pink) or human IgA2 control mAb (light pink) for 1 h. Subsequently, cells were treated with either (C) EVs (7000 EVs/cell), (D) Pam_3_CSK_4_ (TLR2 ligand, 1 ng/mL), or (E) LPS (TLR4 ligand, 1 ng/mL) in the presence of antibodies for 24 h. Cells treated with cell culture medium supplemented only with *Ef*-EVs (7000 EVs/cell) in (C, blue), Pam_3_CSK_4_ (1 ng/mL) in (D, dark blue), or LPS (1 ng/mL) in (E, red) were used as positive activating controls while cells treated with cell culture medium supplemented alone (**C**-**E**, light green) were used as negative controls. NF-κB/AP-1 activation was measured as the activity of secreted SEAP and expressed as a normalized value relative to the positive controls. Data are shown as means ± SD of three independent experiments (*N* = 3, *n* ≥ 3) and analyzed either by Kruskal-Wallis test followed by Dunn’s multiple comparison *post hoc* test in (A and B) or by two-way analysis of variance (ANOVA) followed by Šídák multiple comparison *post hoc* test in (**C**-**E**)

We first studied whether *Ef*-EVs could induce pro-inflammatory responses in human macrophages by quantifying NF-κB/AP-1 activation in dTHP1-XBlue cells in response to *Ef*-EVs from the type strain *E. faecalis* DSM 20478 at different concentrations (100 − 50,000 EVs per cell). Our results demonstrated that *Ef-*EVs triggered the activation of the NF-κB/AP-1 pathways in human macrophages, following a dose-dependent pattern (Fig. 3A). Concentrations of 5000 EVs per cell and higher led to a statistically significant increase in NF-κB/AP-1 activity compared to the untreated control. Comparable dose-dependent pro-inflammatory effects were observed when dTHP1-XBlue cells were treated with EVs derived from clinical *E. faecalis* bloodstream isolates (Figs. 3B and S3). Given the comparable activation profiles induced by *Ef*-EVs from bloodstream isolates and the type strain, all subsequent mechanistic studies were conducted using *Ef*-EVs derived from *E. faecalis* DSM 20478.

TLR2 is the principal PRR involved in the recognition of components that assemble the cell wall of Gram-positive bacteria, including LTA and acylated lipoproteins [31, 51]. We therefore investigated whether TLR2 mediates EV-induced inflammation.

As dTHP1-XBlue cells can activate NF-κB/AP-1 *via* activation of a wide array of PRRs, including surface and endosomal TLRs and NLRs, we assessed the dependency of TLR2 signaling during EV-induced inflammation by blocking TLR2 binding sites using a neutralizing antibody before *Ef*-EVs treatment. Cells were treated with varying concentrations of anti-hTLR2-IgA mAb or its isotype control (i.e., human IgA2 control mAb) 1 h before and throughout 24 h of incubation with *Ef*-EVs. As controls, cells were either treated with the TLR2 ligand Pam_3_CSK_4_ or the TLR4 ligand LPS under identical culture conditions. Post-treatment, the effect of TLR2 blockade on stimulus-induced inflammation was assessed by measuring NF-κB/AP-1 activation. As shown in Fig. 3C, NF-κB/AP-1 activation induced by *Ef*-EVs was gradually inhibited by anti-hTLR2-IgA mAb in a similar fashion as for Pam_3_CSK_4_ (Fig. 3D). Conversely, LPS-induced activation remained unaffected regardless of antibody concentration (Fig. 3E). Hence, it indicated that the inflammatory effects of *Ef*-EVs are driven by TLR2 signaling.

### TLR2 is not an endocytic receptor for EV uptake

**Fig. 4:**
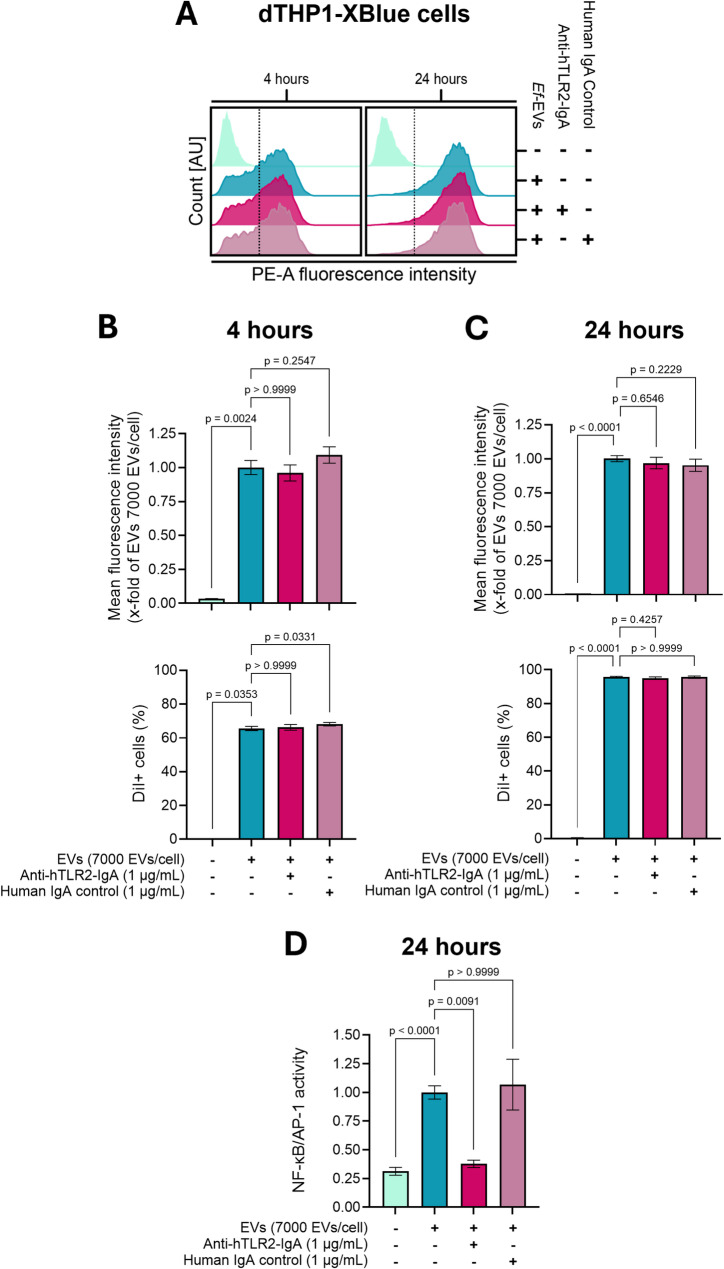
TLR2 controls EV-induced immune activation but does not function as an endocytic receptor for EV uptake. dTHP1-XBlue cells were pretreated with anti-hTLR2-IgA mAb (1 µg/mL) or human IgA2 control mAb (1 µg/mL) for 1 h. Cells were then treated with DiI-labeled *Ef*-EVs (7000 EVs/cell) in the presence of antibodies for 4 and 24 h. Cells incubated in cell culture medium alone were used as negative controls. Cells treated with cell culture medium supplemented with DiI-labeled *Ef*-EVs (7000 EVs/cell) were used as positive activating controls. For EV internalization assays, mean fluorescence intensity (B and C, upper panel) and EV-positive cells (B and C, lower panel) were quantified after 4 (**A** and **B**) and 24 h (**A** and **C**) of EV treatment by measuring fluorescence intensity associated with DiI-labeled *Ef*-EVs on the PE channel. NF-κB/AP-1 activation in D was measured after 24 h of EV incubation as the activity of secreted SEAP and expressed normalized to the positive controls. Quantitative results are presented as mean ± SD (*N* = 3, *n* = 3) and were analyzed by Kruskal-Wallis test followed by Dunn’s multiple comparison *post hoc* test

After identifying TLR2 as the receptor responsible for EV-induced inflammatory signaling, we focused on dissecting its role during EV uptake and the possible connection between EV uptake and immune activation. Consistent with our previous experimental setup, we investigated the role of TLR2 by subjecting dTHP1-XBlue (Fig. 4) and HEK-Dual™ hTLR2 cells (Figure S4) to TLR2 blockade, while simultaneously monitoring EV internalization and NF-κB/AP-1 activation. To this end, cells were treated with anti-hTLR2-IgA mAb or its isotype control antibody for 1 h before and during subsequent incubation with fluorescently labeled *Ef*-EVs (DiI-labeled *Ef*-EVs). Control treatments using SEC-fractionated DiI in PBS (final DiI concentration: 2 µM, DiI control) confirmed the absence of detectable DiI fluorescence (Figure S5), excluding dye-derived artifacts in the uptake assays. EV uptake was monitored after 4 and 24 h, and NF-κB/AP-1 activation was quantified after 24 h of treatment.

As shown in Figs. 4 and S4, EV uptake increased over time, and its extent varied between the reporter cell lines. However, EV internalization and fluorescence intensity was equal among cells treated with anti-hTLR2-IgA mAb, the isotype control, or the control condition (i.e., EVs in cell culture medium), regardless of the analyzed time point (Figs. 4A-C, S4 A-C, and S6). Notably, and consistent with our previous observations, TLR2 blockade reduced the EV-induced NF-κB/AP-1 activation (Figs. 4D and S4D). Consequently, our results implied that while TLR2 recognizes ligands carried by *Ef*-EVs and mediates downstream inflammatory signaling, it does not function as an endocytic receptor for EV uptake.

**Fig. 5:**
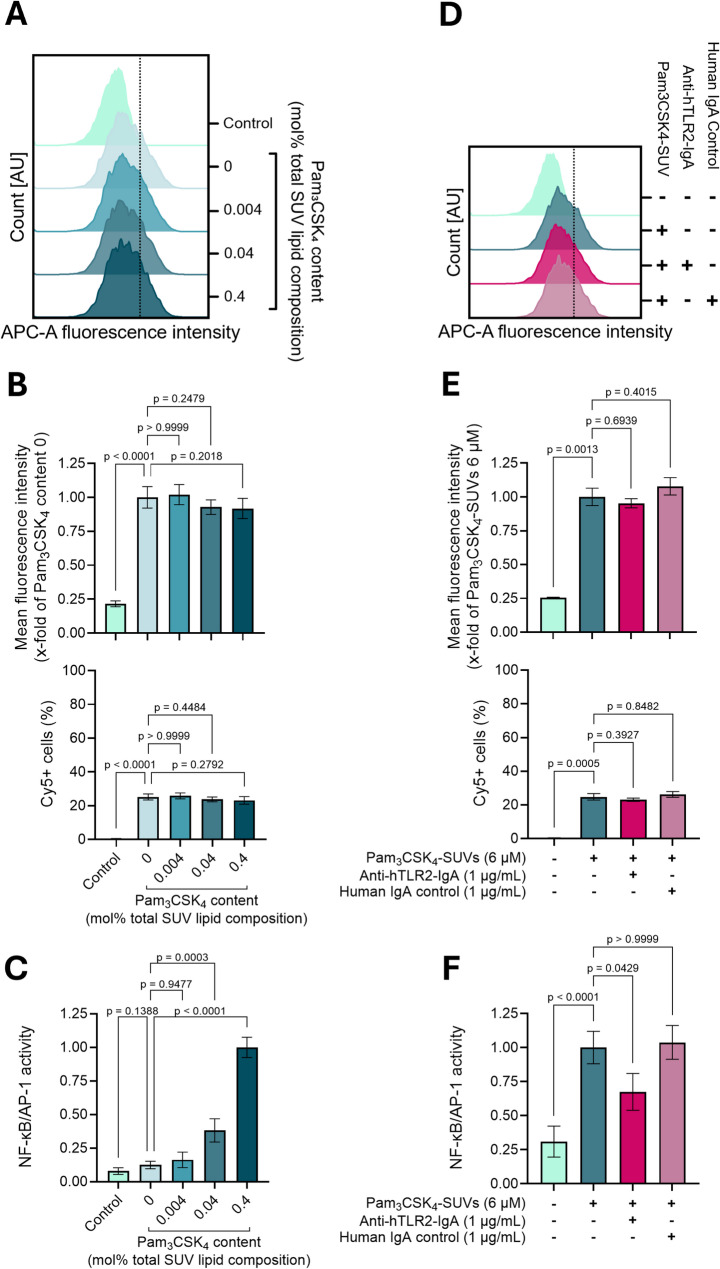
TLR2 engagement by Pam_3_CSK_4_-SUVs triggers inflammatory activation but does not enhance SUV internalization. **A**-**C**) dTHP1-XBlue cells were treated with Pam_3_CSK_4_-SUVs (6 µM) containing increasing amounts of Pam_3_CSK_4_ (from 0 to 0.4 mol% total SUV lipid composition) for 18 h. Cells incubated in cell culture medium alone were used as negative controls. Cells treated with cell culture medium containing Pam_3_CSK_4_-SUVs (6 µM, Pam_3_CSK_4_ composition = 0.4 mol% total SUV lipid composition) were used as positive activating controls. **D**-**F**) dTHP1-XBlue cells were pretreated with anti-hTLR2-IgA mAb (1 µg/mL) or human IgA2 control mAb (1 µg/mL) for 1 h. Cells were then treated with Pam_3_CSK_4_-SUVs (6 µM, Pam_3_CSK_4_ composition = 0.04 mol% total SUV lipid composition) in the presence of antibodies for 18 h. Cells treated with cell culture medium containing Pam_3_CSK_4_-SUVs (6 µM, Pam_3_CSK_4_ composition = 0.04 mol% total SUV lipid composition) were used as positive activating controls. For both experiments, cells incubated in cell culture medium alone were used as negative controls. Mean fluorescence intensity (B and E, upper panel) and SUV-positive cells (B and E, lower panel) were quantified after 18 h of treatment by measuring fluorescence intensity associated with SUVs on the APC channel. In C and F, NF-κB/AP-1 activation was measured after 18 h of SUV treatment as the activity of secreted SEAP and expressed normalized to the positive controls. Quantitative results are shown as mean ± SD (*N* = 3, *n* = 3) and were analyzed by Kruskal-Wallis test followed by Dunn’s multiple comparison *post hoc* test

To further investigate the relationship between TLR2-mediated inflammatory activation and vesicle uptake, we examined the inflammatory effects and internalization dynamics of synthetic EVs, i.e., small unilamellar vesicles (SUVs) formulated with 79 mol% Egg PC, 20 mol% Egg PG, and 1 mol% 18:1 Cyanine 5 PE – to mirror key features of stability and negative charge found in EVs derived from both eukaryotic and prokaryotic cells [45] – and functionalized with Pam_3_CSK_4_ in order to minimally emulate the TLR2 ligand payload of Gram-positive bacterial EVs, hereafter called Pam_3_CSK_4_-SUVs. dTHP1-XBlue cells were treated with 6 µM of Pam_3_CSK_4_-SUVs – designed to resemble *Ef*-EVs in size (average mean size ≈ 150 nm, average mode size ≈ 145 nm; Figure S7) – functionalized with increasing amounts of Pam_3_CSK_4_ (from 0 to 0.4 mol% total SUV lipid composition) for 18 h. Following treatment, both NF-κB/AP-1 activation and SUV uptake were quantified. As shown in Fig. 5C, NF-κB/AP-1 activation increased as a function of Pam_3_CSK_4_ content; however, such differential TLR2 engagement did not translate into differences in SUV internalization across the evaluated conditions (Figs. 5A and B).

### Immune activation is independent of EV internalization

Having identified that TLR2 drives EV-induced inflammatory signaling but does not facilitate EV uptake, we next sought to elucidate the mechanisms of *Ef*-EV uptake and determine whether internalization is functionally linked to immune activation. Accordingly, we investigated the routes of EV uptake in dTHP1-XBlue cells pretreated with pharmacological inhibitors of endocytosis for 30 min and subsequently incubated with DiI-labeled *Ef*-EVs for 4 h in the continued presence of the inhibitors. The concentration of the inhibitors used for this study was determined based on toxicity screening, ensuring minimal adverse effects on cell viability (Figure S8).

**Fig. 6:**
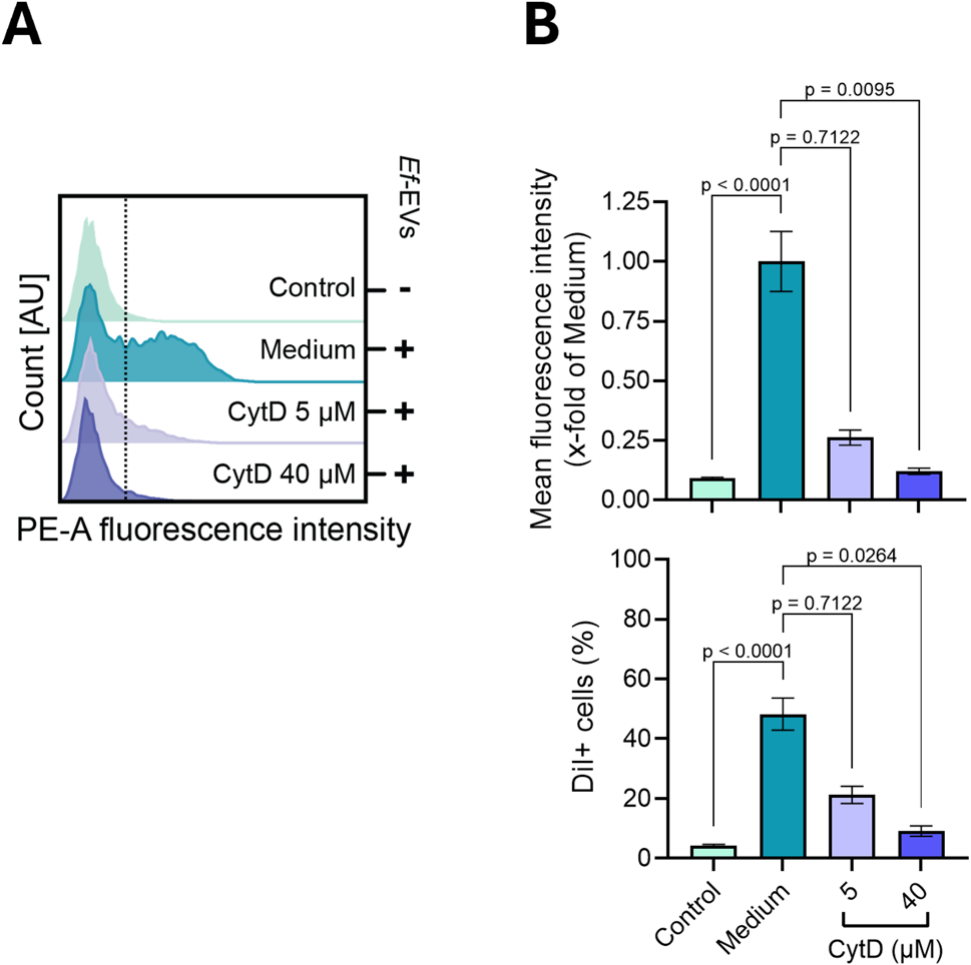
*Ef*-EV internalization depends on actin reorganization. **A** and **B**) dTHP1-XBlue cells were pretreated with CytD (5 µM and 40 µM) for 30 min. Afterward, cells were incubated with DiI-labeled *Ef*-EVs (7000 EVs/cell) in the presence of the inhibitor for 4 h. Mean fluorescence intensity (B, upper panel) and EV-positive cells (B, lower panel) were quantified by measuring fluorescence intensity associated with DiI-labeled *Ef*-EVs on the PE channel. Cells treated by EVs in cell culture medium were used as positive uptake controls. Quantitative results are presented as mean ± SD of three independent experiments (*N* = 3, *n* = 3) and were analyzed by Kruskal-Wallis test followed by Dunn’s multiple comparison *post hoc* test

First, as shown in Fig. 6, we observed that EV internalization was reduced by cytochalasin D (CytD), an inhibitor of actin polymerization, in a dose-dependent manner, being almost completely suppressed at the highest concentration tested. These results indicate that the endocytic mechanisms underlying EV uptake are actin-dependent.

**Fig. 7:**
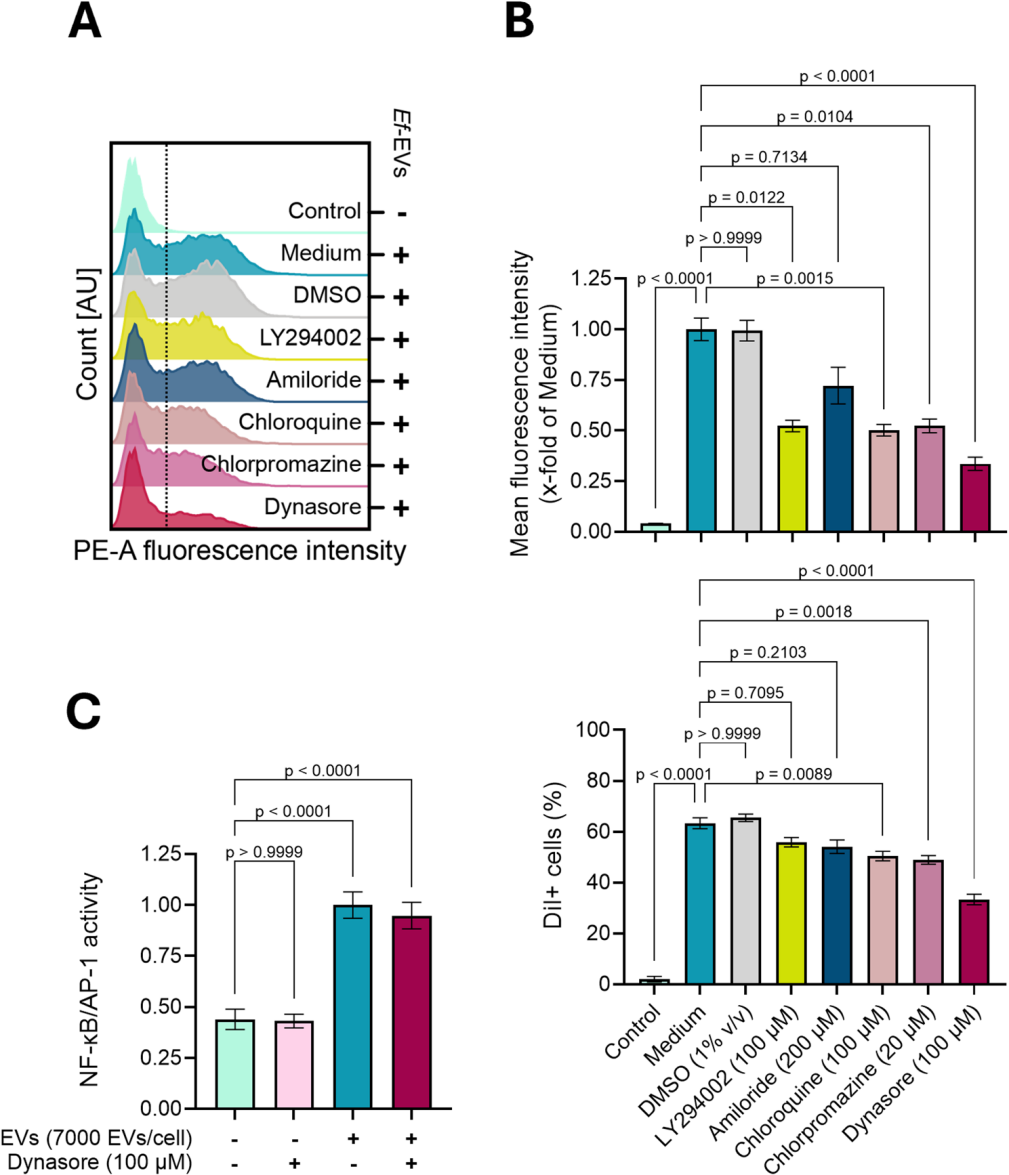
Routes of *Ef*-EV uptake. **A** and **B**) dTHP1-XBlue cells were pretreated with pharmacological endocytosis inhibitors for 30 min. Afterward, cells were incubated with DiI-labeled *Ef*-EVs (7000 EVs/cell) in the presence of inhibitors for 4 h. Mean fluorescence intensity (B, upper panel) and EV-positive cells (B, lower panel) were quantified by measuring fluorescence intensity associated with DiI-labeled *Ef*-EVs on the PE channel. Cells treated by EVs dispersed in cell culture medium or medium containing DMSO (1% v/v) were used as positive uptake controls. **C**) dTHP1-XBlue cells were pretreated with dynasore at 100 µM for 30 min. Afterward, cells were incubated with *Ef*-EVs (7000 EVs/cell) in the presence of dynasore at 100 µM for 24 h. Cells treated with cell culture medium supplemented only with *Ef*-EVs (7000 EVs/cell) were used as positive activating controls. NF-κB/AP-1 activation was measured as the activity of secreted SEAP and expressed as a normalized value relative to the positive control. Quantitative results are presented as mean ± SD of three independent experiments (*N* = 3, *n* ≥ 3) and were analyzed by Kruskal-Wallis test followed by Dunn’s multiple comparison *post hoc* test

We next examined the contribution of specific endocytic routes during EV uptake using pathway-specific inhibitors. As shown in Fig. 7A and B, under control conditions (i.e., EVs in cell culture medium or medium containing DMSO), ~ 60% of cell internalized EVs after 4 h of treatment. Inhibition of phagocytosis and macropinocytosis with LY294002 and amiloride, respectively, resulted in minor (i.e., ⁓55% EV-positive cells) and statistically non-significant reductions in the percentage of EV-positive cells. However, both inhibitors markedly reduced the mean fluorescence intensity compared to control treatments, with LY294002 inducing a two-fold reduction. The inhibition of clathrin-mediated endocytosis by both chloroquine and chlorpromazine reduced EV uptake, resulting in ⁓50% EV-positive cells and a two-fold decrease in the mean fluorescence intensity. Notably, the strongest inhibition of EV uptake was observed following treatment with dynasore, an inhibitor of all dynamin-dependent endocytic pathways, which resulted in a two-fold reduction in the percentage of EV-positive cells and a three-fold decrease in the mean fluorescence intensity. These findings suggest that *Ef*-EVs are internalized through multiple endocytic mechanisms, principally within the spectrum of dynamin-dependent pathways.

To investigate whether EV internalization is required for immune activation, we next assessed NF-κB/AP-1 activity in dTHP1-XBlue cells after 24 h *Ef*-EV treatment in the presence of dynasore. As shown in Fig. 7C, the inhibition of EV uptake did not affect NF-κB/AP-1 activation. These results implied that immune activation induced by *Ef*-EVs is independent of EV endocytosis, suggesting that inflammatory signaling is initiated at the cell surface of host macrophages upon receptor engagement. As dTHP1-XBlue cells express multiple PRRs, uptake inhibition experiments were interpreted as assessing the requirement for vesicle internalization per se, rather than involvement of receptor-specific signaling pathways.

### Transcriptomic analyses of macrophages derived from EV-treated zebrafish larvae revealed the functional impact of *Ef*-EVs on inflammation and metabolism

Following our observations on the in vitro pro-inflammatory effects of *Ef*-EVs, we examined the impact of EVs on host innate immunity using zebrafish larvae as a relevant 3R-compatible in vivo model system [52]. *Ef*-EVs were injected into the yolk sac of 3-dpf zebrafish larvae, and their effects on the transcriptomic profile of macrophages were assessed by RNA-Seq analysis. No signs of toxicity were observed in zebrafish larvae injected with *Ef*-EVs or PBS, i.e., the control group (Figure S9). Green-fluorescent embryonic zebrafish macrophages were isolated by FACS 18 h post-injection from both EV-treated and control groups for downstream analysis.

**Fig. 8:**
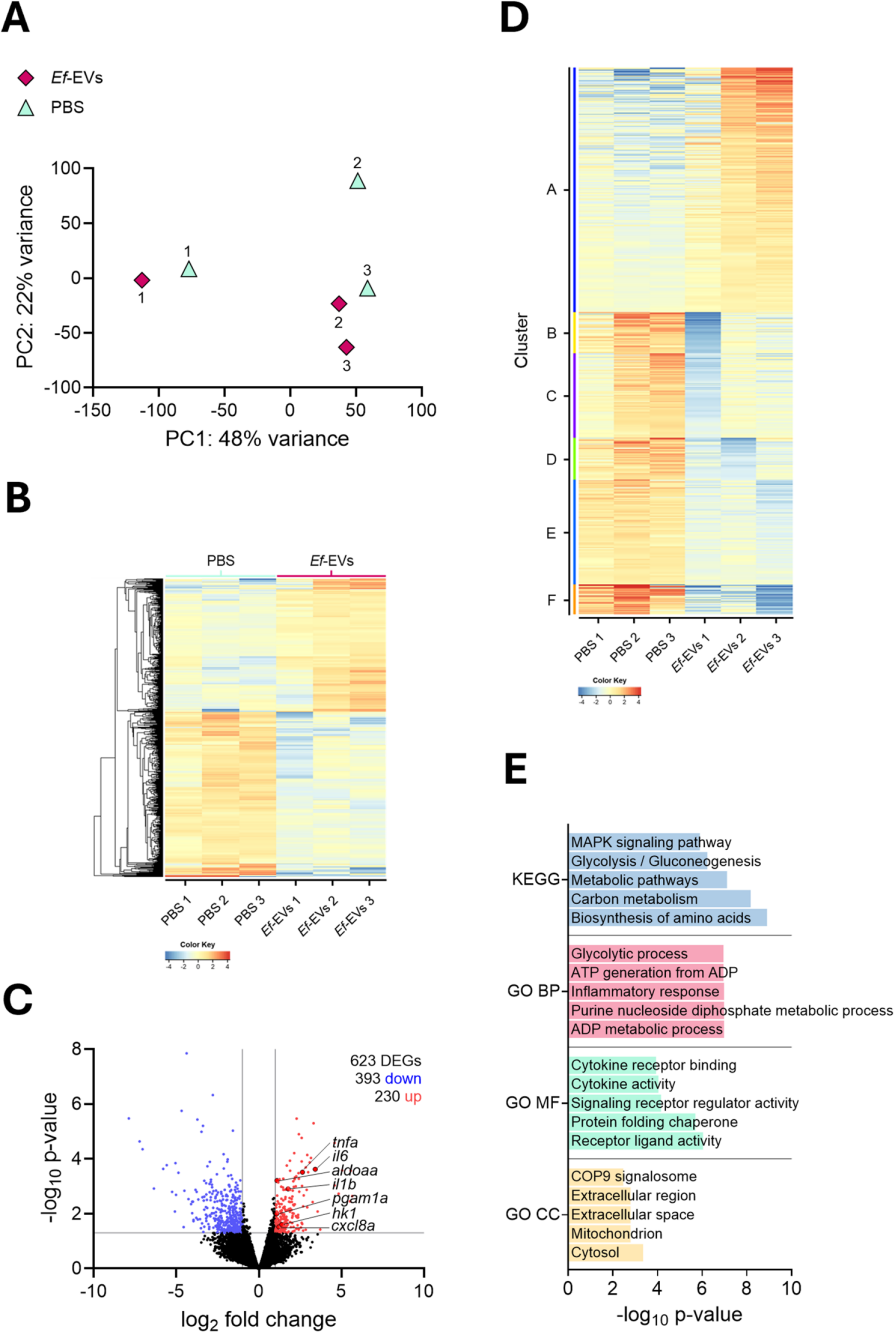
mRNA sequencing transcriptome profiling of zebrafish ex vivo embryonic macrophages following in vivo EVs zebrafish larvae treatment (*N* = 3). **A** Principal component analysis of *Ef*-EV or PBS-treated zebrafish macrophages. Each dot represents one independent biological preparation. **B** Heat map displaying DEGs across individual samples, with hierarchical clustering highlighting patterns of gene expression. **C** Volcano plot showing the distribution of DEGs, with 230 upregulated (red) and 393 downregulated (blue) genes in macrophages after *in vivo Ef*-EV treatment. Log_2_ fold change is plotted against -log_10_ *p*-values. **D** k-means clustering and enrichment analysis for DEGs. **E** Selected KEGG pathways and GO terms for biological processes (GO BP), molecular functions (GO MF), and cellular components (GO CC) from Cluster A, showing key metabolic and signaling pathways. Data used for analysis are reported in Tables S1–S4

Principal component analysis confirmed a separation between the experimental groups, with principal components (PCs) 1 and 2 accounting for 48% and 22% of the variance, respectively (Fig. 8A). Hierarchical clustering of differentially expressed genes (DEGs) demonstrated distinct gene expression profiles between the experimental groups, as visualized in the heatmap (Fig. 8B). 623 DEGs were identified (fold change > 2, and p < 0.05), of which 230 were upregulated, and 393 were downregulated in the Ef-EV group compared to PBS controls (Fig. 8C). Among the upregulated genes were the pro-inflammatory cytokines tnf, il6, and il1b; the chemokine cxcl8a; and key metabolic genes involved in glycolysis, including aldoaa, pgam1a, and hk1. TPM values for DEGs were used for hierarchical k-means clustering (Fig. 8D). Analysis of enriched pathways of genes from Cluster A – the largest resulting gene cluster – revealed the activation of key metabolic and inflammatory pathways (Figure 8E). Specifically, Kyoto Encyclopedia of Genes and Genomes (KEGG) pathways related to glycolysis/gluconeogenesis, as well as the Gene Ontology terms for biological processes (GO BP) for glycolytic process and inflammatory response, were significantly upregulated. Therefore, our results indicate that the effects of Ef-EVs are not limited to inflammatory activation but also extend to metabolic reprogramming.

### *Ef*-EVs shape inflammatory and metabolic responses in primary human macrophages

To examine whether the previously observed immunomodulatory properties of *Ef*-EVs are conserved in a biologically relevant human context, we next investigated the effects of EVs on primary human macrophages. First, the cytotoxic properties of *Ef*-EVs were evaluated in human monocyte-derived macrophages (HMDMs) by quantifying cell viability using the MTT assay after 24-hour treatment with varying concentrations of EVs (1000-10,000 EVs/cell). As a result, no reduction in cell viability was observed relative to the control, suggesting that *Ef*-EVs do not induce cytotoxicity in primary human macrophages (Figure S10A).

**Fig. 9:**
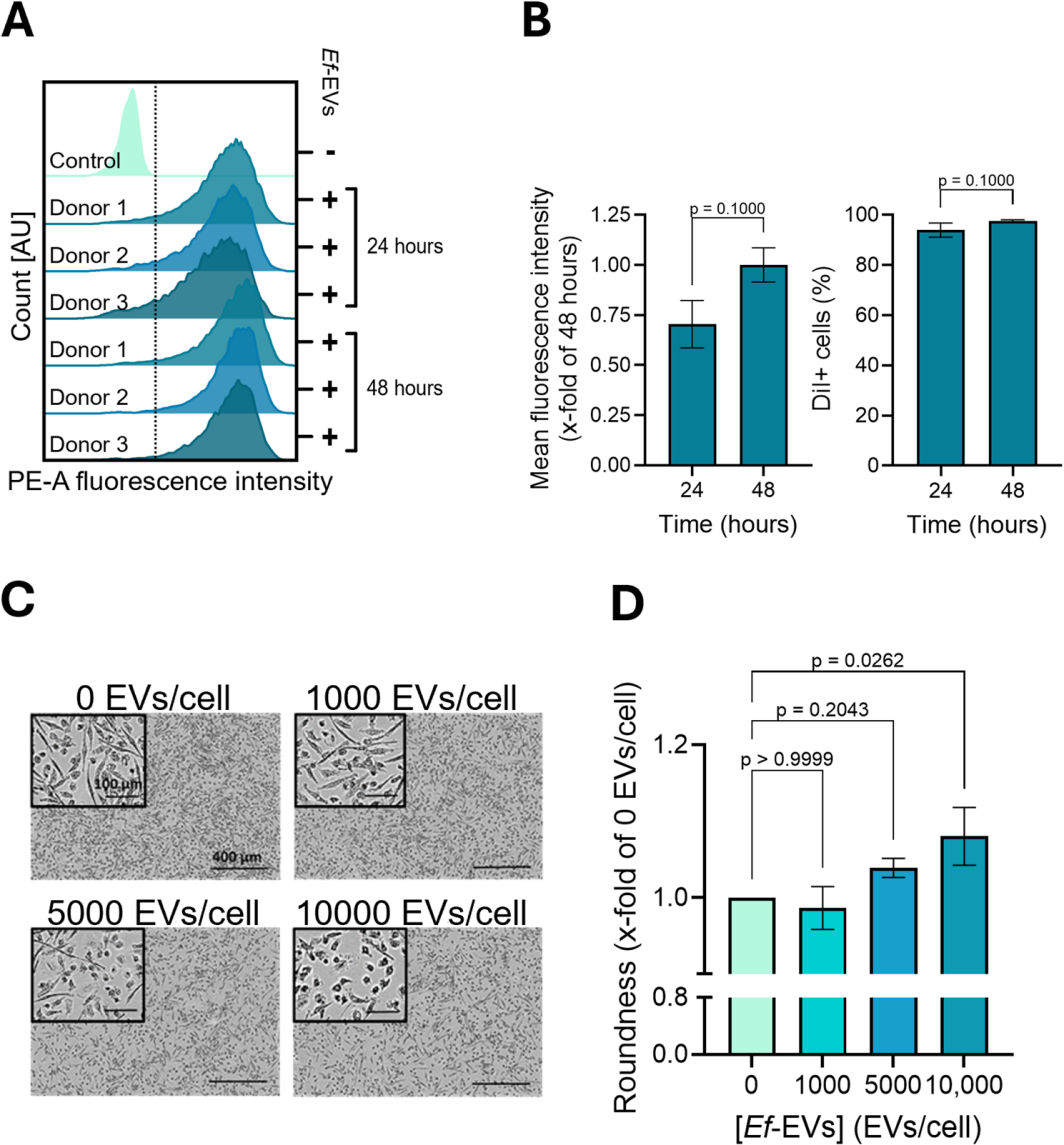
*Ef*-EVs are internalized by primary human macrophages and modulate their morphology. **A** and **B**) HMDMs were incubated with DiI-labeled *Ef*-EVs (30,000 EVs/cell) for 24 and 48 h. Mean fluorescence intensity (B, left panel) and EV-positive cells (B, right panel) were quantified by measuring fluorescence intensity associated with DiI-labeled *Ef*-EVs on the PE channel. Results are presented as mean ± SD of three individual donors (*N* = 3, *n* = 1). **C** Representative images of macrophages treated with *Ef*-EVs (1000-10,000 EVs/cell) for 24 h (scale bar in full micrograph = 400 μm, scale bar in zoomed micrograph = 100 μm). **D** X-fold change of roundness compared to medium-treated cells (0 EVs/cell). Statistical comparisons of two groups in B were performed by Mann-Whitney test. Results in D were analyzed by Kruskal-Wallis test followed by Dunn’s multiple comparison *post hoc* test

We next studied the potential role of *Ef*-EVs as vehicles for the intracellular delivery of bioactive cargo, assessing whether host macrophages internalized them. To this end, HMDMs were treated with DiI-labeled *Ef*-EVs, and EV uptake was quantified after 24 and 48 h of incubation. We observed that primary macrophages actively endocytosed EVs, with ⁓95% of cells internalizing them within the first 24 h of treatment (Figs. 9A and B). Furthermore, the percentage of cells that internalized EVs, as well as the mean fluorescence intensity, showed a trend of increase after 48 h of incubation, suggesting a time-dependent EV uptake. Aligned with our previous results on EV-induced activation of inflammatory signaling, we examined whether *Ef*-EVs skew primary human macrophages toward a pro-inflammatory state. As extensively documented [42, 49, 53], macrophages adopt distinct morphologies depending on their polarization status, with round cells indicating an inflammatory phenotype. Leveraging this effect, the ability of *Ef*-EV to modulate macrophage polarization was assessed by monitoring morphological changes of HMDMs after 24 h of EV treatments. Our data indicate that *Ef*-EVs promote a round-shaped phenotype in macrophages, i.e., pro-inflammatory polarization, in a dose-dependent manner, with higher EV concentrations leading to a more pronounced cell rounding (Figs. 9C and D).

**Fig. 10:**
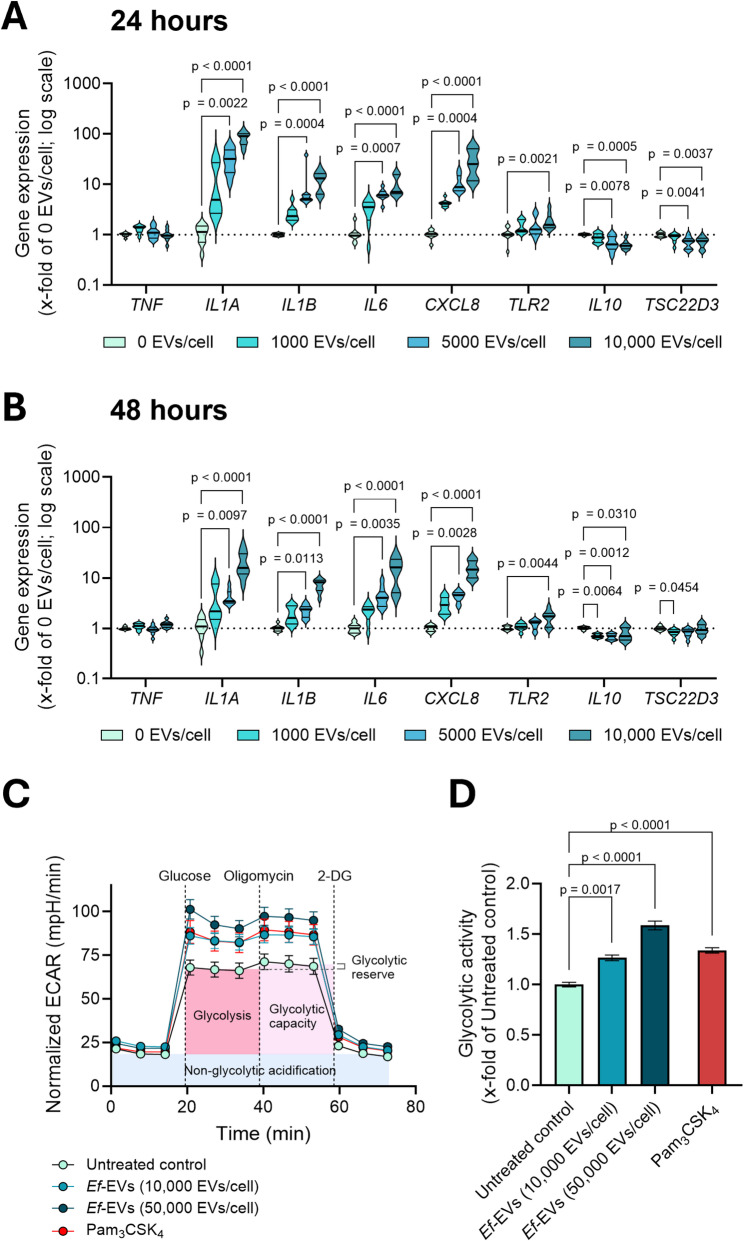
*Ef*-EVs modulate gene expression and metabolic activity in primary human macrophages. **A** and **B**) HMDMs were incubated with *Ef*-EVs at different concentrations (1000-10,000 EVs/cell) for 24 (A) and 48 h (B). Data are presented as frequency distributions, median, and quartiles of gene expression results from three individual donors (*N* = 3, *n* = 3) and normalized to medium-treated cells (0 EVs/cell) as control. C and D) HMDMs were treated with *Ef*-EVs (10,000 and 50,000 EVs/cell) or Pam_3_CSK_4_ (10 ng/mL) for 24 h. Cells incubated in cell culture medium alone served as untreated control. **C** Normalized ECAR values were monitored after injections of glucose, oligomycin, and 2-DG, according to the glycolysis stress test. **D** Glycolytic activity is shown as the x-fold change of untreated control regarding the normalized ECAR values in glycolysis. Results C and D are shown as mean ± SEM of three individual donors (*N* = 3, *n* = 6). Results were analyzed by Kruskal-Wallis test followed by Dunn’s multiple comparison *post hoc* test

Macrophage pro-inflammatory polarization was further analyzed by monitoring the changes in the expression of pro- and anti-inflammatory genes in HMDMs within 24 and 48 h of EV treatments. Consistent with our previous results, our data identified a significant dose-dependent upregulation of the gene expression of pro-inflammatory cytokines, such as interleukin (IL)-1α, IL-1β, IL-6, and IL-8 (gene name *CXCL8*) in the first 24 h of EV treatment (Fig. 10A). The gene expression of anti-inflammatory IL-10 and glucocorticoid-induced leucine zipper (GILZ, gene name *TSC22D3* [54–57]) was significantly downregulated compared to the control group. Although to a lower extent, a similar expression pattern was observed after 48 h of EV treatment (Fig. 10B). Expression of TLR2 mRNA showed a significant increase after 24 h (Fig. 10A) in the group with the highest EV concentration (10,000 EVs/cell). This effect persisted after 48 h (Fig. 10B). On the other hand, the expression of *TNF* remained relatively stable, showing a slight increase in the group with the least EV concentration (1000 EVs/cell) after 24 h (Fig. 10A) and in the group with the higher EV concentration (10,000 EVs/cell) after 48 h (Fig. 10B). To assess whether the glycolysis-promoting properties of *Ef*-EVs observed in vivo in zebrafish larvae translate into metabolic reprogramming in primary human macrophages, we assessed the glycolytic function of HMDMs following 24-hour treatment with *Ef*-EVs using the glycolysis stress test. Cells treated with Pam_3_CSK_4_ or left untreated served as controls. As shown in Fig. 10C, extracellular acidification rate (ECAR) measurements revealed that macrophages treated with *Ef*-EVs displayed a marked increase in glycolytic activity compared to the untreated control. This effect was dose-dependent and closely mirrored the metabolic response induced by Pam_3_CSK_4_ (Figs. 10C and D). Taken together, these findings demonstrate that *Ef*-EVs promote a metabolic shift towards aerobic glycolysis in host macrophages, consistent with the induction of a pro-inflammatory phenotype.

### *Ef*-EVs induce inflammatory responses in primary human endothelial cells

**Fig. 11:**
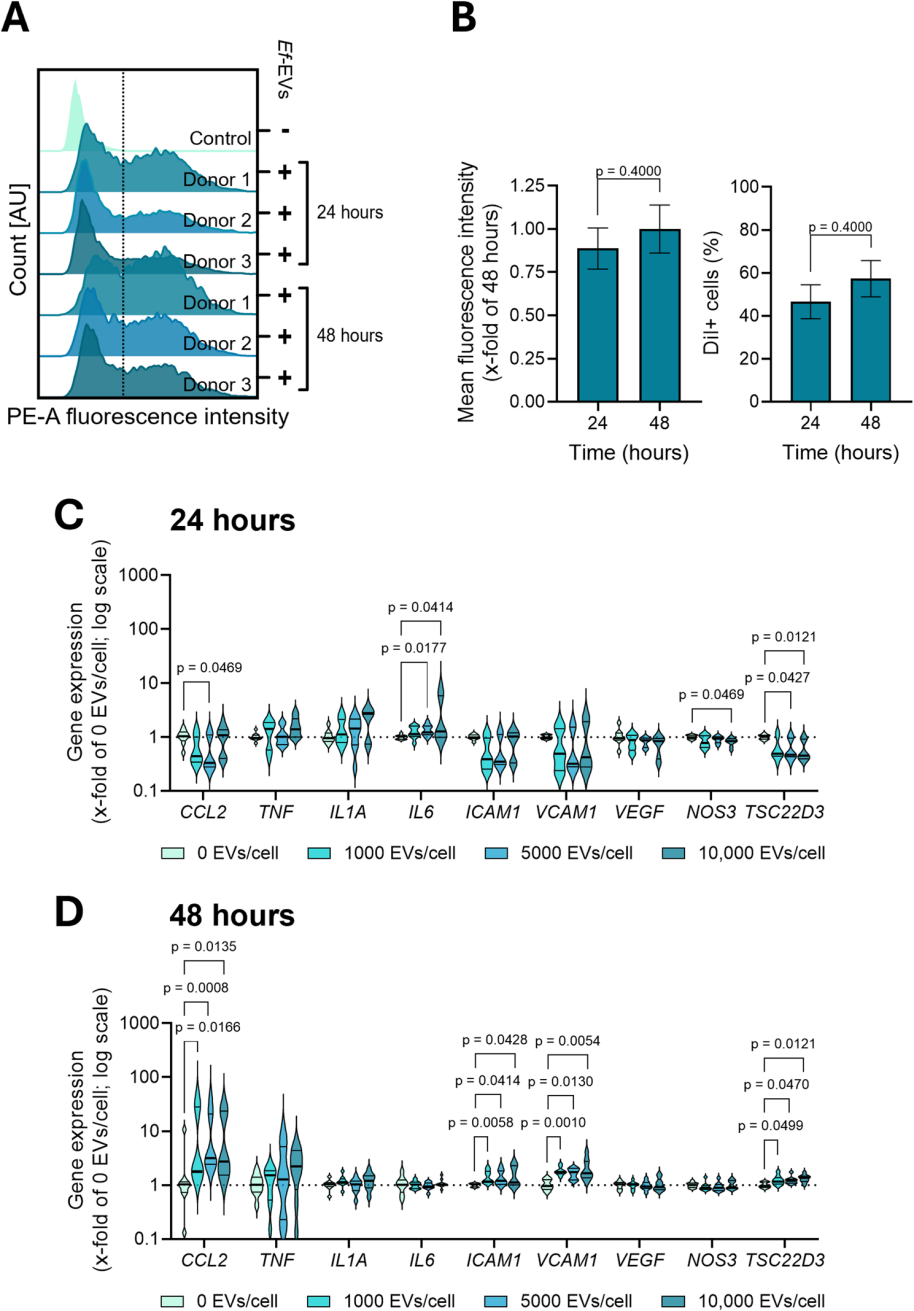
*Ef*-EVs are internalized by primary human endothelial cells and modulate their gene expression. **A** and **B**) HUVECs were incubated with DiI-labeled *Ef*-EVs (30,000 EVs/cell) for 24 and 48 h. Mean fluorescence intensity (B, left panel) and EV-positive cells (B, right panel) were quantified by measuring fluorescence intensity associated with DiI-labeled *Ef*-EVs on the PE channel. Results are presented as mean ± SD of three individual donors (*N* = 3, *n* = 1). **C** and **D**) HUVECs were incubated with *Ef*-EVs at different concentrations (1000-10,000 EVs/cell) for 24 (C) and 48 h (D). Data are presented as frequency distributions, median, and quartiles of gene expression results from three individual donors (*N* = 3, *n* = 3) and normalized to medium-treated cells (0 EVs/cell) as control. Statistical comparisons of two groups in B were performed by Mann-Whitney test. Results in C and D were analyzed by Kruskal-Wallis test followed by Dunn’s multiple comparison *post hoc* test

Growing evidence has shown that bacteria-derived EVs can cross host barriers and access the systemic cirulation, allowing bacteria to influence the course of pathophysiological processes remotely [33–36, 58]. In this context, we evaluated the biological effects of *Ef*-EVs on primary human endothelial cells. Quantifications of cell viability by MTT assay of human umbilical vein endothelial cells (HUVECs) after 24-hour treatments with varying EV concentrations (1000-10,000 EVs/cell) did not show any significant reduction in cell viability (Figure S10B), indicating that *Ef*-EVs were not cytotoxic under the tested conditions. Following cell treatments with DiI-labeled *Ef*-EVs, we observed that HUVECs actively internalized EVs (Figs. 11A and B). Specifically, ⁓45% of treated cells endocytosed EVs after 24 h of treatment. In a similar manner to HMDMs, EV internalization and mean fluorescence intensity tended to increase after 48 h of incubation, suggesting a time-dependent uptake of EVs. Monitoring levels of gene expression after 24 and 48 h of EV treatment, we found that *Ef*-EVs modulate gene expression in a similar trend as in HMDMs, namely, EVs upregulated the expression levels of pro-inflammatory genes and reduced the abundance of mRNAs encoding for anti-inflammatory proteins (Fig. 11C and D). Specifically, the levels of *IL6* mRNA experienced a significant dose-dependent increase after 24 h of EV treatment (Fig. 11C). However, such upregulations returned to control levels at the later time point (Fig. 11D). Reductions in the expression of anti-inflammatory endothelial nitric oxide synthase (eNOS, gene name *NOS3*) and GILZ (*TSC22D3*) were observed after 24 h of treatment (Fig. 11C). Gene expression of monocyte chemoattractant protein-1 (MCP-1, gene name *CCL2*), intercellular adhesion molecule-1 (*ICAM1*), and vascular cell adhesion molecule-1 (*VCAM1*) increased after 48 h (Fig. 11D). These results suggest that, although to a lower extent as compared to HMDMs, *Ef*-EVs can modulate inflammatory responses of human primary endothelial cells, potentially contributing to endothelial dysfunction.

## Discussion

*E. faecalis* frequently causes bacteremia, endocarditis, and sepsis, reflecting its ability to colonize the bloodstream and evade host defenses [1–4, 59]. While research over the past few decades has clarified the function and relevance of enterococcal virulence factors during disease progression [1], the overall mechanisms behind bacterial colonization, immunoevasion, and immunosuppression of their hosts need to be further understood [3, 11, 17].

Interkingdom crosstalk between host and microbiome *via* EVs is a widely observed phenomenon. Indeed, EVs derived from both commensal and pathogenic bacteria can cross host barriers [58, 60, 61] through paracellular or transcellular pathways [33]. As such, bacteria-derived EVs have been detected suspended in biological fluids, including blood [62] and urine [63, 64]. Although trafficking dynamics differ depending on bacterial species [33], EVs protect and transport biomolecules to sites beyond the GI tract [34], influencing immune and metabolic processes in distal organs independently of direct bacterial colonization [33, 58]. Consequently, numerous studies have reported the possible mechanistic participation of bacteria-derived EVs in the development of different diseases, including inflammatory bowel disease (IBD), Alzheimer’s disease, metabolic syndrome, and atherosclerosis [58, 61, 62, 65–67]. Imperatively, it remains to be resolved whether bacteria-derived EVs contribute to the preparation of niches for bacterial colonization in distant tissues in the host [68].

Due to their cell wall structure, it was long assumed that biogenesis and release of EVs would not occur in Gram-positive bacteria. Consequently, the biological relevance of Gram-positive bacterial EVs has been understudied [20, 25, 27]. While mechanisms regulating vesiculogenesis remain poorly understood [19], the formation of EVs is currently viewed as a universal phenomenon that originates from the budding of the cytoplasmic membrane in regions differentially enriched with fatty acids and phospholipids and whose release relies on membrane fluidity and cell wall integrity [19, 25].

On this basis, we have investigated the immunomodulatory effects of EVs derived from *Enterococcus faecalis* on host cells. We identified *Ef*-EVs as modulators of host immune responses in vitro and in vivo, triggering pro-inflammatory responses *via* TLR2 signaling without inducing cytotoxicity. We observed that *Ef*-EVs are readily internalized by host cells, with dynamin-mediated endocytic pathways serving as major entry routes. While TLR2 activation by Gram-positive bacterial components is well established [31, 51], our data provide mechanistic insight into how *Ef*-EVs engage this pathway, demonstrating that receptor activation can occur independently of vesicle internalization. Identified by the transcriptomic analysis of macrophages isolated from EV-treated zebrafish larvae and further confirmed in primary human macrophages, our results indicate that *Ef*-EVs shift the cellular metabolism toward glycolysis.

Research on *E. faecalis-*derived EVs remains limited. Following different *E. faecalis* culture conditions and EV isolation strategies, previous studies have reported that the particle size of *Ef*-EVs falls from 20 nm to 400 nm [38, 39, 69–71] – a size range commonly observed in other Gram-positive bacterial EVs [19, 25, 72]. As reported by Costantini et al., the size of non-purified *Ef*-EVs obtained by ultracentrifugation (UC) of overnight *E. faecalis* DSM 20478 culture supernatants ranged from ~ 180 nm to ~ 210 nm [69]. A slightly downshifted particle size distribution profile was identified by Ma et al. in non-purified *Ef*-EV preparations obtained by UC of previously concentrated overnight *E. faecalis* DSM 2570 culture supernatant, with an EV mean and mode size of 165.5 nm and 165.1 nm, respectively [39]. Similarly, and consistent with our data, Afonina et al. observed that the size of *Ef*-EVs isolated by OptiPrep density gradient fractionation of a concentrated *E. faecalis* OG1RF culture supernatants harvested at the late exponential growth phase ranged between 50 nm and 400 nm, with a particle size distribution centered at ~ 100 nm [38]. Likewise, our results were not limited to the evaluation of non-purified EVs but rather correspond to the characterization of *Ef*-EVs purified by SEC – a mild isolation process that does not compromise the biological activity or integrity of EVs [73]. Interestingly, environmental conditions have also been shown to influence *Ef*-EV size. Niu et al. reported the isolation of EVs from 12-hour *E. faecalis* OG1RF cultures grown at pH 7.4 or pH 9.0. Following EV isolation by UC and SEC, *Ef*-EVs ranged from 50 to 400 nm, with a mean diameter of 199.69 ± 11.41 nm at pH 7.4 compared to 134.86 ± 15.76 nm at pH 9.0, indicating that alkaline conditions promote the production of smaller EVs [70]. Likewise, Chan et al. reported that EVs isolated from 10-hour *E. faecalis* OG1RF cultures grown at pH 7.0 or pH 9.0, using a protocol involving supernatant concentration, UC, and OptiPrep density gradient fractionation, had mean particle sizes of 139.9 ± 67.0 nm and 129.5 ± 75.9 nm, respectively, supporting the conclusion that alkaline environments favor the formation of smaller *Ef*-EVs [71].

Shed from the bacterial cytoplasmic membrane, bacteria-derived EVs are constituted of a varied pro-inflammatory payload (i.e., PAMPs) derived from their parent bacterium, including soluble and membrane-associated proteins, lipids, and nucleic acids [20, 58, 74]. In turn, recognized by PRRs (e.g., TLRs and NLRs) on host cells, EV cargo can induce downstream signaling pathways and exert similar pro-inflammatory responses as the ones observed by the whole bacterium [19, 28–30].

Previous studies have demonstrated that infection with *E. faecalis* induces a pro-inflammatory shift in the phenotype of murine macrophages [15, 75, 76]. In detail, Mohamed Elashiry et al. reported that *E. faecalis*-infected macrophages skewed their polarization towards a pro-inflammatory phenotype, as evidenced by an increased expression of CD38 and IRF5 compared to non-infected control groups [15]. Likewise, Tien et al. observed that NF-κB reporter murine macrophage-like cells (RAW-Blue™ cells) activated NF-κB as a result of either *E. faecalis* infection at a low Multiplicity of Infection (MOI, MOI = 1 and 10), exposure to heat-killed *E. faecalis*, or treatment with supernatants from bacterial cultures [75]. Using bone marrow-derived macrophages (BMDMs), Zou et al. reported that macrophage infection by the *E. faecalis* E99 strain significantly increased the expression levels of cytokines associated with macrophages in a pro-inflammatory state (e.g., TNF, IL-1β, and INF-γ), primarily driven by NF-κB and dependent on physical contact between bacteria and macrophages [76]. In line with the known immunostimulatory properties of *E. faecalis*, we found that *Ef*-EVs alone can trigger pro-inflammatory polarization in primary human macrophages. Despite the methodological variability described above regarding *Ef*-EV isolation, most studies report harvesting EVs from anaerobic *E. faecalis* cultures at different growth phases [38, 39, 70, 71]. Across these reports, *Ef*-EVs are consistently described as potent inducers of pro-inflammatory responses, promoting the polarization of macrophage cell lines toward a pro-inflammatory phenotype and being associated with the upregulation of inflammatory mediators, including NF-κB, TNF, IL-1β, and IL-6 [38, 39, 70, 71]. Notably, Niu et al. described that *Ef*-EVs derived from *E. faecalis* cultures grown under alkaline conditions (pH 9.0) exhibit enhanced pro-inflammatory activity compared to EVs obtained at neutral pH [70]. Complementary, *Ef*-EVs harvested from aerobically cultured *E. faecalis* have been reported to exert antiviral effects, reducing HIV-1 replication in human T-lymphocyte MT-4 cells [69]. Together, these findings suggest that culture conditions, including oxygen availability and pH, may influence the immunomodulatory properties of *Ef*-EVs.

TLR2, being the principal PRR involved in the recognition of components from the cell wall of Gram-positive bacteria [31, 51], mediates the inflammatory effects induced by *Ef*-EVs, as evidenced by the inhibition of this effect using anti-hTLR2 antibodies. As previously reported by Afonina et al., structure characterization of *Ef*-EV indicates an enrichment of lipoproteins [38], similar to those identified in EVs from other Gram-positive bacterial species, with a known effect activating the transcription factor NF-κB through TLR1/TLR6-TLR2 heterodimers [77–79]. For instance, treatments with either mutant strains of *Listeria* deficient in functional lipoproteins or their culture supernatants resulted in decreased TLR2-mediated NF-κB activity [80].

The inflammatory activity observed for *Ef*-EVs is comparable to reports describing pro-inflammatory effects of EVs derived from Gram-negative bacteria whose immunostimulatory activity is primarily mediated by TLR2 signaling. *Burkholderia pseudomallei* and *Helicobacter pylori* have been shown to activate inflammatory responses through TLR2-dependent mechanisms due to the presence of atypical LPS structures that function as TLR2 agonists [81–84]. EVs derived from *B. pseudomallei* exhibit adjuvant properties, promoting murine dendritic cell (DC) activation in vitro and in vivo, as evidenced by the upregulation of maturation and co-stimulatory markers, along with increased pro-inflammatory cytokine secretion [85]. Similarly, *H. pylori*-derived EVs have been shown to induce inflammatory responses *via* NF-κB activation [86, 87], even in the absence of TLR4 engagement [86]. Moreover, in the absence of canonical TLR4-binding LPS, EVs derived from the endotoxin-free *E. coli* strain ClearColi BL21(DE3) display suppressed TLR4-driven NF-κB activation but retained a robust TLR2 activity due to the presence of TLR2 agonists [88].

Despite its role in initiating inflammatory signaling, our results demonstrate that TLR2 does not act as an endocytic receptor for EV uptake. Indeed, using SUVs functionalized with Pam_3_CSK_4_ as a reductionist EV model [40] to exclusively assess the role of TLR2 ligand payload present in native Gram-positive bacterial EVs, we demonstrated that TLR2 engagement and EV uptake are uncoupled processes. Studies available in this regard present conflicting findings, and no consensus has been established. For instance, Brandt et al. observed a reduced NF-κB activity in HEK-Blue™-hTLR2 cells treated with non-internalizable beads conjugated to TLR2 ligands (LTA, and Pam_3_CSK_4_), reporting that inflammatory activation requires the internalization of membrane-anchored TLR2 *via* clathrin- and dynamin-dependent endocytic pathways [32]. In agreement with our findings, Oosenbrug et al. found that THP1-Dual™ cells activated NF-κB after cell incubation in plates containing immobilized TLR2 ligand Pam_2_CSK_4_, concluding that TLR2-dependent pro-inflammatory signaling originates from the cell surface in monocytic cells [89]. Shamsul et al. observed that the TLR2 ligand FSL-1 could be internalized by peritoneal macrophages from TLR2-deficient mice, demonstrating that it is taken up by macrophages via a clathrin-dependent endocytic pathway mediated by the TLR2 accessory molecules CD14 and CD36 [90]. Besides these results, Trianiafilou et al. observed that the TLR2 ligand LTA internalizes CD14-transfected HEK293 cells without TLR2, stating that TLR2 ligand-induced activation occurs at the plasma membrane and subsequent trafficking is independent of signaling [91]. Comparable to these results, Müller et al. observed in primary murine keratinocytes that the TLR2 ligand SitC colocalized with TLR2 and stimulated the expression of proinflammatory cytokines and intracellular TLR2 [92]. However, SitC was internalized into TLR2 knockout cells, demonstrating that its uptake is TLR2-independent [92]. Interestingly, Shen et al. identified that the molecular mechanisms underlying cross-presentation of the lipidated TLR2 agonist cytotoxic T lymphocyte (CTL) epitope Pam_2_IDG were mediated by TLR2 [93]. Specifically, TLR2 facilitates antigen uptake in bone marrow-derived dendritic cells (BMDCs) through a mechanism that is entirely dependent on clathrin-mediated endocytosis [93]. Therefore, although our findings did not support the role of TLR2 as an endocytic receptor for EVs, it may play an important role in the presentation of EV-derived antigens. 

Our findings identified dynamin-dependent endocytic routes as the primary mechanisms for *Ef*-EV internalization. Similar observations have been reported by Wang et al. in EVs derived from other Gram-positive bacteria. In their study, it was shown that human macrophages internalized EVs isolated from a community-associated methicillin-resistant *Staphylococcus aureus* strain, and this process was significantly reduced by inhibiting dynamin-dependent endocytosis with Dynasore [94]. As in our study, the residual EV uptake observed by Wang et al. may be attributed to the involvement of multiple entry routes influenced by additional attributes, such as EV size [58]. Although *Ef*-EVs displayed a narrow size distribution, our EV suspensions contained a heterogeneous population of particles. In turn, size heterogeneity may enable *Ef*-EVs to utilize multiple uptake pathways – a phenomenon also observed in EVs derived from Gram-negative bacteria [58]. Cellular internalization dynamics of *Ef*-EVs differed from previous observations in extracellular vesicles derived from eukaryotic cells [49]. In detail, Mashayekhi et al. reported that both macropinocytosis and phagocytosis drive endocytosis of carboxyfluorescein succinimidyl ester (CFSE)-labeled EVs derived from HCT116 colorectal cancer cells, specifying that the inhibition of dynamin-dependent pathways has a minimal impact on EV uptake [49]. Taken together, it suggests that the mechanisms of cellular uptake that EVs exploit are dependent on their biogenic source [26].

Complementarily, our data demonstrate that *Ef*-EV-induced NF-κB/AP-1 activation can be initiated independently of vesicle internalization. This conclusion is supported by the observation that pharmacological inhibition of EV uptake reduced vesicle internalization but did not diminish NF-κB/AP-1 activation. In parallel, TLR2 blockade significantly attenuated NF-κB/AP-1 activation without affecting EV uptake, supporting a membrane-proximal mechanism of signal initiation. Importantly, our analysis is restricted to the early TLR2-dependent NF-κB/AP-1 signaling axis investigated in this study and does not exclude additional internalization-dependent pathways that may contribute to *Ef*-EV-mediated immune responses in other cellular contexts or at later time points [21, 39].

Connected to inducing pro-inflammatory activation, this study demonstrates that *Ef*-EVs rewire host cell metabolism. Transcriptomic analysis of macrophages isolated from zebrafish larvae treated with *Ef*-EV revealed the upregulation of genes involved in key cellular functions, such as glycolysis and gluconeogenesis, as well as inflammatory responses. In line with these findings, we observed that *Ef*-EVs promoted a metabolic switch toward glycolysis, as evidenced by a dose-dependent increase in extracellular acidification rate. This metabolic reprogramming is a hallmark of classically activated pro-inflammatory macrophages, which undergo a transition from oxidative phosphorylation to aerobic glycolysis upon inflammatory stimulation [95, 96]. Fleetwood et al. reported similar metabolic reprogramming effects induced by bacterial EVs. Investigating immunostimulatory properties of EVs isolated from the Gram-negative bacterium *Porphyromonas gingivalis*, the authors demonstrated that these EVs not only activate host immune and inflammatory responses but also drive a metabolic transition from oxidative phosphorylation to glycolysis in both human monocyte-derived and murine bone-marrow-derived macrophages [97]. This heightened glycolysis supports the energetic and biosynthetic demands associated with membrane remodeling and the production and secretion of pro-inflammatory mediators, including TNF-α, IL-1β, nitric oxide, and reactive oxygen species as response to immunological insults [96].

A limitation of the present work is that inflammatory signaling was primarily evaluated through NF-κB/AP-1 activation, using human macrophage-like cells as a well-established reductionist model to investigate PRR-mediated innate immune responses. TLR signaling can proceed via MyD88-dependent or TRIF-dependent pathways, which drive pro-inflammatory cytokine production and type I interferon induction, respectively [98, 99]. TLR2, as a MyD88-dependent receptor [31], can activate multiple downstream signaling cascades in addition to NF-κB, including mitogen-activated protein kinase (MAPK) pathways [31, 100], interferon regulatory factors (IRFs)-mediated responses [100], inflammasome activation [101], and signal transducer and activator of transcription (STAT) signaling [31], all of which contribute to the regulation of inflammatory responses. However, given its role as a central regulator of TLR signaling and its well-established relevance in macrophage activation [102], NF-κB activation provides a biologically meaningful readout of pro-inflammatory properties of *Ef*-EVs. While our findings provide mechanistic insight into *Ef*-EV-induced TLR2 signaling, they do not aim to fully recapitulate the complexity of in vivo host responses, which involve a highly regulated participation of multiple cell types, including neutrophils, epithelial cells, and adaptive immune populations. Therefore, future studies expanding EV-induced immune modulation analysis to other signaling axes and incorporating additional immune cell types and more complex model systems will be important to further define the physiological relevance of these findings.

## Conclusion

Throughout this study, we provide novel insights into the immunomodulatory and metabolic effects, as well as facilitated signaling mechanisms of *E. faecalis*-derived extracellular vesicles on host cells. We demonstrate that *Ef*-EVs induce a pro-inflammatory phenotype *via* TLR2-dependent signaling. Despite their engagement with TLR2, our findings reveal that TLR2 does not act as an endocytic receptor during EV uptake, which primarily occurs *via* dynamin-dependent mechanisms. We confirmed that immune activation induced by *Ef*-EVs originates at the plasma membrane upon TLR2 anchoring, independently of EV uptake. Coupled to immune activation, *Ef*-EVs induce metabolic reprogramming toward glycolysis in host macrophages.

Taken together, our study identifies *Ef*-EVs as active immunomodulatory agents that influence both innate immune signaling and cellular metabolism. These findings highlight the mechanism that Gram-positive bacterial EVs use for shaping host-pathogen interactions and suggest that EVs contribute to the systemic impact of *E. faecalis* infections. Future studies should clarify their diagnostic value and evaluate their potential as targets for immunomodulation.

## Supplementary Information

Supplementary Material 1.

Supplementary Material 2: Figure S1: Growth curve of clinical E. faecalis bloodstream isolates and E. faecalis DSM 20478. The optical density (OD600) was measured from E. faecalis cultures in BHI medium grown under static conditions at 37 °C. Results are shown as mean ± SD (N = 3, n = 3). Figure S2: Isolation and characterization of EVs derived from clinical E. faecalis bloodstream isolates and E. faecalis DSM 20478. (Left panel) Representative chromatogram obtained by protein concentration analysis of the first 20 eluted fractions after size exclusion chromatography for each bacteria strain. Protein concentration was quantified by the BCA assay. Results are shown as mean ± SD (N = 1, n = 3). (Right panel) Representative size distribution of particles in the vesicle-richest fraction by Nanoparticle Tracking Analysis. Figure S3: Pro-inflammatory effects of EVs derived from clinical E. faecalis bloodstream isolates and E. faecalis DSM 20478. dTHP1-XBlue cells were treated with EVs derived from clinical E. faecalis bloodstream isolates (1000-10,000 EVs/cell). LPS (100 ng/mL) and Pam3CSK4 (100 ng/mL) were used as positive controls. After 4 hours of treatment, the concentration of TNF and the combined concentration of IL-1α and IL-1β secreted in the cell culture supernatants were quantified using HEK-Blue™ TNF-α and HEK-Blue™ IL-1R cells, respectively. Cytokine concentrations were determined by interpolation from a standard curve, generated with recombinant human TNF (1 pg/mL - 10 ng/mL) or recombinant human IL-1β (0.01 pg/mL - 10 ng/mL). Data are shown as means ± SD of three independent experiments (N = 3, n = 3) and analyzed by Kruskal-Wallis test followed by Dunn’s multiple comparison post hoc test. Figure S4. TLR2 controls EV-induced immune activation but does not function as an endocytic receptor for EV uptake. HEK-Dual™ hTLR2 cells were pretreated with anti-hTLR2-IgA mAb (1 µg/mL) or human IgA2 control mAb (1 µg/mL) for 1 hour. Cells were then treated with DiI-labeled Ef-EVs (7000 EVs/cell) in the presence of antibodies for 4 and 24 hours. Cells incubated in cell culture medium alone were used as negative controls. Cells treated with cell culture medium supplemented with DiI-labeled Ef-EVs (7000 EVs/cell) were used as positive activating controls. For EV internalization assays, mean fluorescence intensity (B and C, upper panel) and EV-positive cells (B and C, lower panel) were quantified after 4 (A and B) and 24 hours (A and C) of EV treatment by measuring fluorescence intensity associated with DiI-labeled Ef-EVs on the PE channel. NF-κB/AP-1 activation in D was measured after 24 hours of EV incubation as the activity of secreted SEAP and expressed normalized to the positive controls. Quantitative results are presented as mean ± SD (N = 3, n = 3) and were analyzed by Kruskal-Wallis test followed by Dunn’s multiple comparison post hoc test. Figure S5: A and B) dTHP1-XBlue cells were treated with a control EV-mock solution (DiI control) consisting of SEC-fractionated DiI in PBS (final DiI concentration: 2 µM, DiI control) and DiI-labeled Ef-EVs (7000 EVs/cell). Cells incubated in cell culture medium alone were used as negative controls. Cells treated with cell culture medium supplemented with DiI-labeled Ef-EVs (7000 EVs/cell) were used as positive controls. Mean fluorescence intensity (B, upper panel) and EV-positive cells (B, lower panel) were quantified after 4 hours of treatment by measuring fluorescence intensity associated with DiI-labeled Ef-EVs on the PE channel. Results are presented as mean ± SD (N = 3, n = 3) and were analyzed by Kruskal-Wallis test followed by Dunn’s multiple comparison post hoc test. Figure S6: TLR2 does not function as an endocytic receptor for EV uptake. dTHP1-XBlue cells were pretreated with anti-hTLR2-IgA mAb (1 µg/mL) or human IgA2 control mAb (1 µg/mL) for 1 hour. Cells were then treated with Ef-EVs (7000 EVs/cell) in the presence of antibodies for 24 hours. Confocal micrographs present cell membrane (WGA-FITC panel), cell nuclei (DAPI panel), and fluorescence associated with DiI-labeled Ef-EVs (DiI panel) independently and merged (Merge panel) (scale bar = 50 µm). Figure S7: Particle size characterization of Pam3CSK4-SUVs as measured by NTA. A) Average mean size and B) average mode size of SUVs containing increasing amounts of Pam3CSK4 (from 0 to 0.4 mol% total SUV lipid composition). Results are presented as mean ± SD (N = 3, n = 3) and were analyzed by Kruskal-Wallis test followed by Dunn’s multiple comparison post hoc test. Figure S8: Toxicity screening of pharmacological inhibitors of endocytosis in dTHP1-XBlue cells. Cells were treated with different amounts of A) amiloride (macropinocytosis inhibitor, from 20 µM to 200 µM), B) chloroquine (clathrin-mediated endocytosis inhibitor, from 10 µM to 100 µM), C) chlorpromazine (clathrin-mediated endocytosis inhibitor, from 10 µM to 100 µM), D) dynasore (dynamin-dependent endocytosis inhibitor, from 20 µM to 200 µM), E) LY294002 (phagocytosis inhibitor, from 10 µM to 100 µM), and F) Cytochalasin (actin polymerization inhibitor, from 0.1 µM to 40 µM). After 4.5 hours of treatment, cell viability was assessed using the MTT assay. Results are shown as mean ± SD (N = 3, n = 3). Figure S9: Kaplan-Meier graph shows the percentage of survival up to 48 hours post-injection with Ef-EVs and PBS. Zebrafish larvae were injected with either 4 nL Ef-EVs (200,000 EVs) or 4 nL PBS at the 3rd-dpf into the yolk sac (N = 3, n = 20). Results indicate the number of live larvae was monitored for 48 hours post-injection. Figure S10: HMDMs (A) and HUVECs (B) were incubated with Ef-EVs (1000-10,000 EVs/cell). After 24 hours of incubation, cell viability was assessed using the MTT assay. Results are shown as mean ± SD of three individual donors (N = 3, n = 3) for HMDMs and mean ± SD of two individual donors (N = 2, n = 6) for HUVECs. Results were analyzed by Kruskal-Wallis test followed by Dunn’s multiple comparison post hoc test.

## Data Availability

The mRNA-Seq raw and processed data were stored in the GEO database (GEO accession number: GSE304960).

## Abbreviations

2-DG: 2-Deoxy-D-glucose
3Rs: Replacement, reduction, and refinement
ACTB: Actin beta
ANOVA: Analysis of variance
AP-1: Activator protein 1
APC: Allophycocyanin
BCA: Bicinchoninic acid
BHI: Brain heart infusion
BMDCs: Bone marrow–derived dendritic cells
BP: Biological processes
BSA: Bovine serum albumin
CC: Cellular components
CD: Cluster of differentiation
cryo-TEM: Cryogenic transmission electron microscopy
CTL: Cytotoxic T lymphocyte
Cyanine 5 PE: 1,2-dioleoyl-sn-glycero-3-phosphoethanolamine-N-(Cyanine 5)
DAPI: 4′,6-Diamidino-2-phenylindole dihydrochloride
DEGs: Differentially expressed genes
DGE: Differential gene expression
DMEM: Dulbecco’s Modified Eagle Medium
DMSO: Dimethyl sulfoxide
dpf: Day post fertilization
DSMZ: German Collection of Microorganisms and Cell Cultures
DTT: Dithiothreitol
ECAR: Extracellular acidification rate
EDTA: Ethylenediaminetetraacetic acid
Ef-EVs: E. faecalis-derived EVs
Egg PC: Egg L-α-phosphatidylcholine
Egg PG: Egg L-α-phosphatidylglycerol
eNOS: Endothelial nitric oxide synthase
EVs: Extracellular vesicles
FACS: Fluorescence‑activated cell sorting
FCS: Fetal calf serum
FITC: Fluorescein isothiocyanate
FSC: Forward scatter
FSL-1: Fibroblast-stimulating lipopeptide-1
GEO: Gene Expression Omnibus
GFP: Green fluorescent protein
GI track: Gastrointestinal tract
GILZ: Glucocorticoid-induced leucine zipper
GO: Gene Ontology
HEPES: N-2-hydroxyethylpiperazine-N-2-ethane sulfonic acid
HMDMs: Human monocyte-derived macrophages
HUVECs: Human umbilical vein endothelial cells
ICAM1: Intercellular adhesion molecule-1
IFN: Interferon
IL: Interleukin
KEGG: Kyoto Encyclopedia of Genes and Genomes
LPS: Lipopolysaccharides
LTA: Lipoteichoic acid
MCP-1: Monocyte chemoattractant protein-1
M-CSF: Macrophage colony-stimulating factor
MF: Molecular functions
MTC: Maximum tolerated concentration
MTT: 3-(4,5-dimethylthiazol-2-yl)-2,5-diphenyltetrazolium bromide
NF-κB: Nuclear factor kappa-light-chain-enhancer of activated B cells
NLR: NOD-like receptor
NOD: Nucleotide-binding oligomerization domain
NTA: Nanoparticle tracking analysis
OD: Optical density
Pam_3_CSK_4_: Synthetic bacterial lipopeptide Pam_3_-Cys-Ser-Lys_4_
PAMPs: Pathogen-associated molecular patterns
PBMCs: Peripheral blood mononuclear cells
PBS: Phosphate buffered saline
PC: Principal components
PCA: Principal Component Analysis
PE: Phycoerythrin
PFA: Paraformaldehyde
PGNs: Peptidoglycans
PMA: Phorbol 12-myristate-13-acetate
PRRs: Pattern recognition receptors
PVDF: Polyvinylidene fluoride
RPMI: Roswell Park Memorial Institute
SD: Standard deviation
SEAP: Secreted embryonic alkaline phosphatase
SEC: Size exclusion chromatography
SEM: Standard error of the mean
SSC: Side scatter
SUVs: Small unilamellar vesicles
TLR: Toll-like receptor
TNF: Tumor necrosis factor alpha
TNFR: Tumor necrosis factor receptor
TPM: Transcripts per kilobase million
UC: Ultracentrifugation
VCAM1: Vascular cell adhesion molecule-1
VEGFA: Vascular endothelial growth factor
WGA: Wheat germ agglutinin
