# Supplementary material for: Extracellular vesicles derived from Enterococcus faecalis: inflammatory activation does not require internalization
Source: Cell Commun Signal. 2026 May 18;24:301. doi: 10.1186/s12964-026-02926-9 (PMC13196059; doi:10.1186/s12964-026-02926-9)
Supplement: Supplementary file 2 — Supplementary Material 2: Figure S1: Growth curve of clinical E. faecalis bloodstream isolates and E. faecalis DSM 20478. The optical density (OD600) was measured from E. faecalis cultures in BHI medium grown under static conditions at 37 °C. Results are shown as mean ± SD (N = 3, n = 3). Figure S2: Isolation and characterization of EVs derived from clinical E. faecalis bloodstream isolates and E. faecalis DSM 20478. (Left panel) Representative chromatogram obtained by protein concentration analysis of the first 20 eluted fractions after size exclusion chromatography for each bacteria strain. Protein concentration was quantified by the BCA assay. Results are shown as mean ± SD (N = 1, n = 3). (Right panel) Representative size distribution of particles in the vesicle-richest fraction by Nanoparticle Tracking Analysis. Figure S3: Pro-inflammatory effects of EVs derived from clinical E. faecalis bloodstream isolates and E. faecalis DSM 20478. dTHP1-XBlue cells were treated with EVs derived from clinical E. faecalis bloodstream isolates (1000-10,000 EVs/cell). LPS (100 ng/mL) and Pam3CSK4 (100 ng/mL) were used as positive controls. After 4 hours of treatment, the concentration of TNF and the combined concentration of IL-1α and IL-1β secreted in the cell culture supernatants were quantified using HEK-Blue™ TNF-α and HEK-Blue™ IL-1R cells, respectively. Cytokine concentrations were determined by interpolation from a standard curve, generated with recombinant human TNF (1 pg/mL - 10 ng/mL) or recombinant human IL-1β (0.01 pg/mL - 10 ng/mL). Data are shown as means ± SD of three independent experiments (N = 3, n = 3) and analyzed by Kruskal-Wallis test followed by Dunn’s multiple comparison post hoc test. Figure S4. TLR2 controls EV-induced immune activation but does not function as an endocytic receptor for EV uptake. HEK-Dual™ hTLR2 cells were pretreated with anti-hTLR2-IgA mAb (1 µg/mL) or human IgA2 control mAb (1 µg/mL) for 1 hour. Cells were then treated with DiI-labe [file 12964_2026_2926_MOESM2_ESM.docx]

# Supplementary results


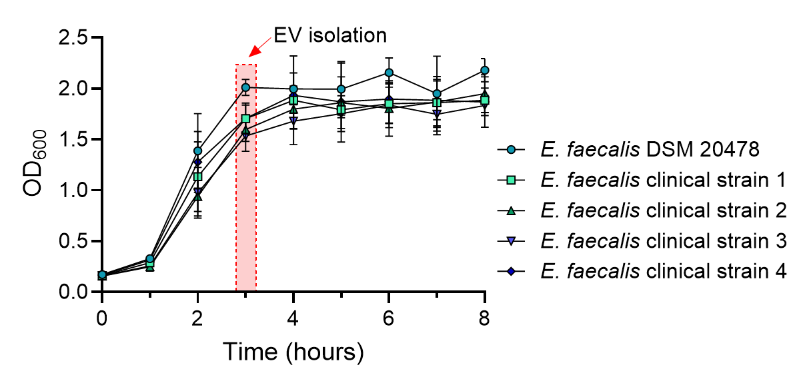


**Figure S1**: Growth curve of clinical *E. faecalis* bloodstream isolates and *E. faecalis* DSM 20478. The optical density (OD_600_) was measured from *E. faecalis* cultures in BHI medium grown under static conditions at 37 °C. Results are shown as mean ± SD (N = 3, n = 3).


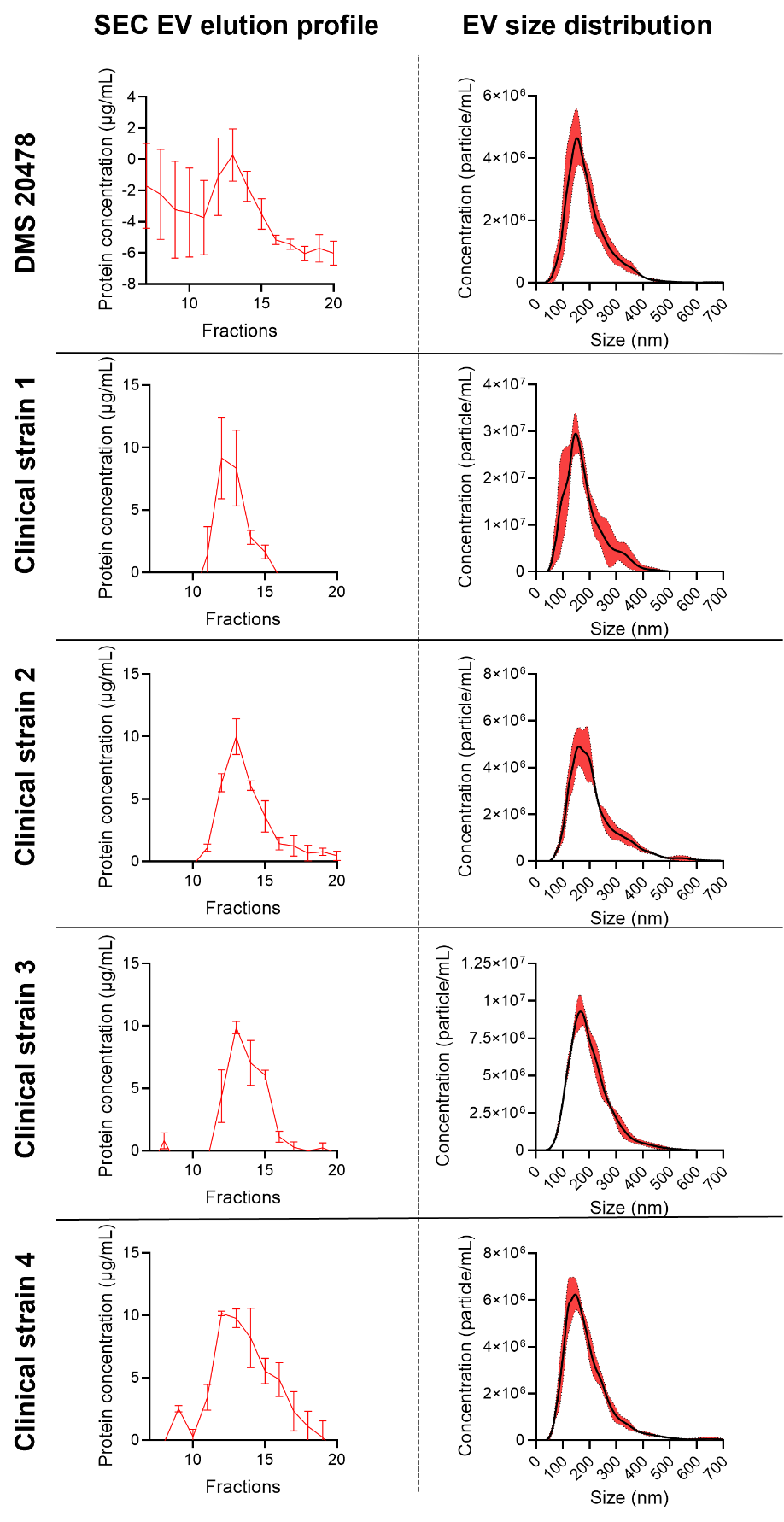


**Figure S2**: Isolation and characterization of EVs derived from clinical *E. faecalis* bloodstream isolates and *E. faecalis* DSM 20478. (Left panel) Representative chromatogram obtained by protein concentration analysis of the first 20 eluted fractions after size exclusion chromatography for each bacteria strain. Protein concentration was quantified by the BCA assay. Results are shown as mean ± SD (N = 1, n = 3). (Right panel) Representative size distribution of particles in the vesicle-richest fraction by Nanoparticle Tracking Analysis.


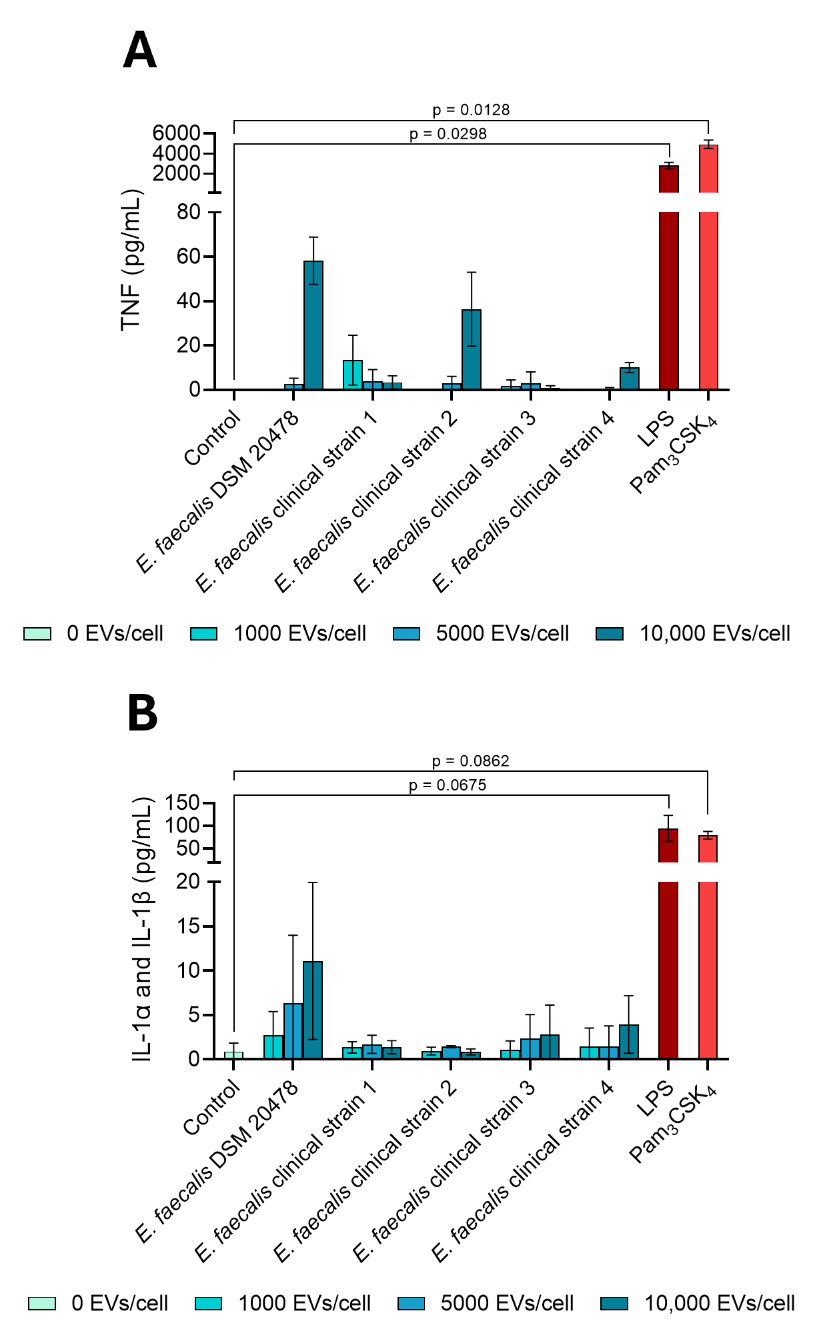


**Figure S3**: Pro-inflammatory effects of EVs derived from clinical *E. faecalis* bloodstream isolates and *E. faecalis* DSM 20478. dTHP1-XBlue cells were treated with EVs derived from clinical *E. faecalis* bloodstream isolates (1000-10,000 EVs/cell). LPS (100 ng/mL) and Pam_3_CSK_4_ (100 ng/mL) were used as positive controls. After 4 hours of treatment, the concentration of TNF and the combined concentration of IL-1α and IL-1β secreted in the cell culture supernatants were quantified using HEK-Blue™ TNF-α and HEK-Blue™ IL-1R cells, respectively. Cytokine concentrations were determined by interpolation from a standard curve, generated with recombinant human TNF (1 pg/mL - 10 ng/mL) or recombinant human IL-1β (0.01 pg/mL - 10 ng/mL). Data are shown as means ± SD of three independent experiments (N = 3, n = 3) and analyzed by Kruskal-Wallis test followed by Dunn’s multiple comparison *post hoc* test.


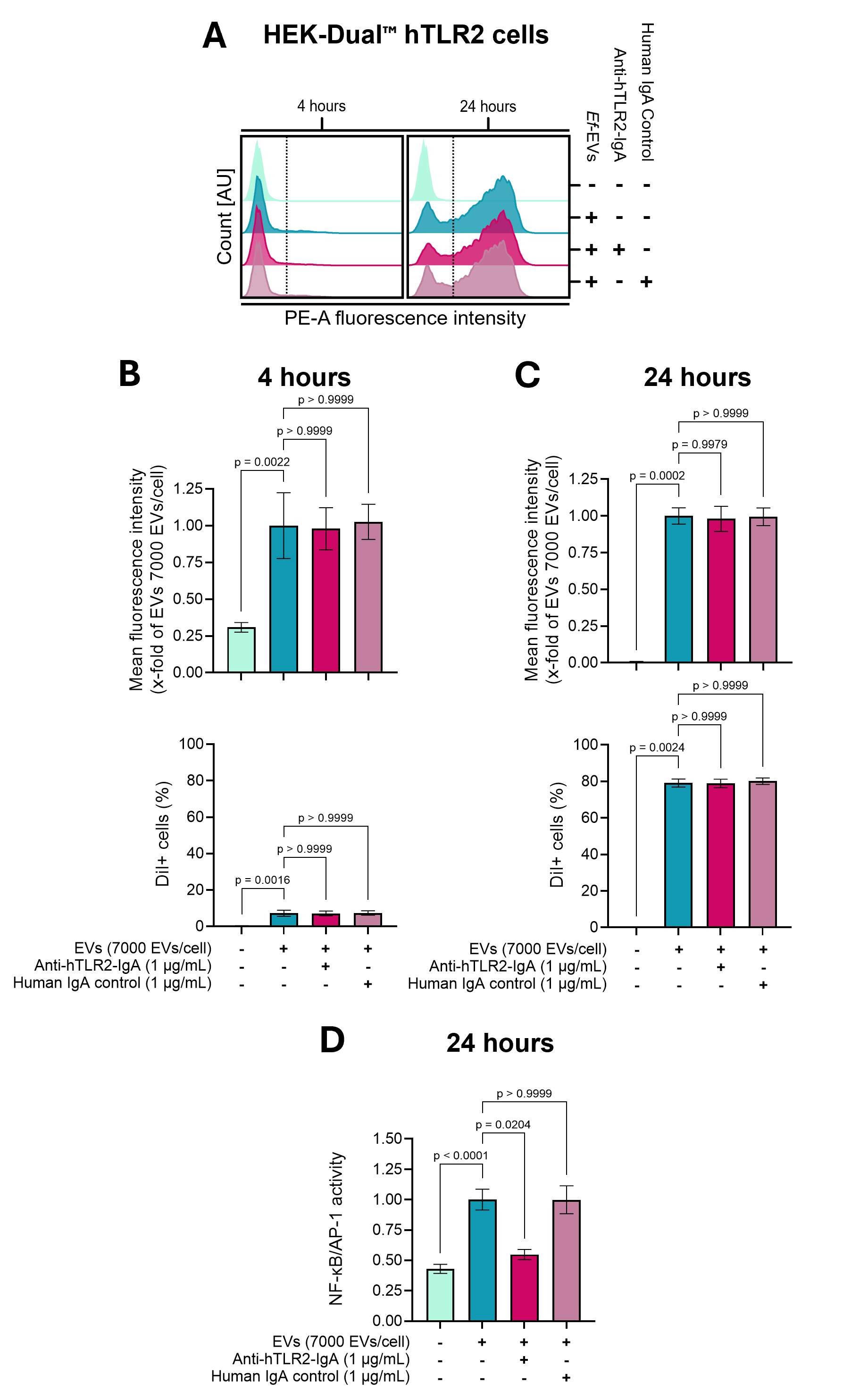


**Figure S4**. TLR2 controls EV-induced immune activation but does not function as an endocytic receptor for EV uptake. HEK-Dual™ hTLR2 cells were pretreated with anti-hTLR2-IgA mAb (1 µg/mL) or human IgA2 control mAb (1 µg/mL) for 1 hour. Cells were then treated with DiI-labeled *Ef*-EVs (7000 EVs/cell) in the presence of antibodies for 4 and 24 hours. Cells incubated in cell culture medium alone were used as negative controls. Cells treated with cell culture medium supplemented with DiI-labeled *Ef*-EVs (7000 EVs/cell) were used as positive activating controls. For EV internalization assays, mean fluorescence intensity (B and C, upper panel) and EV-positive cells (B and C, lower panel) were quantified after 4 (A and B) and 24 hours (A and C) of EV treatment by measuring fluorescence intensity associated with DiI-labeled *Ef*-EVs on the PE channel. NF-κB/AP-1 activation in D was measured after 24 hours of EV incubation as the activity of secreted SEAP and expressed normalized to the positive controls. Quantitative results are presented as mean ± SD (N = 3, n = 3) and were analyzed by Kruskal-Wallis test followed by Dunn’s multiple comparison *post hoc* test.


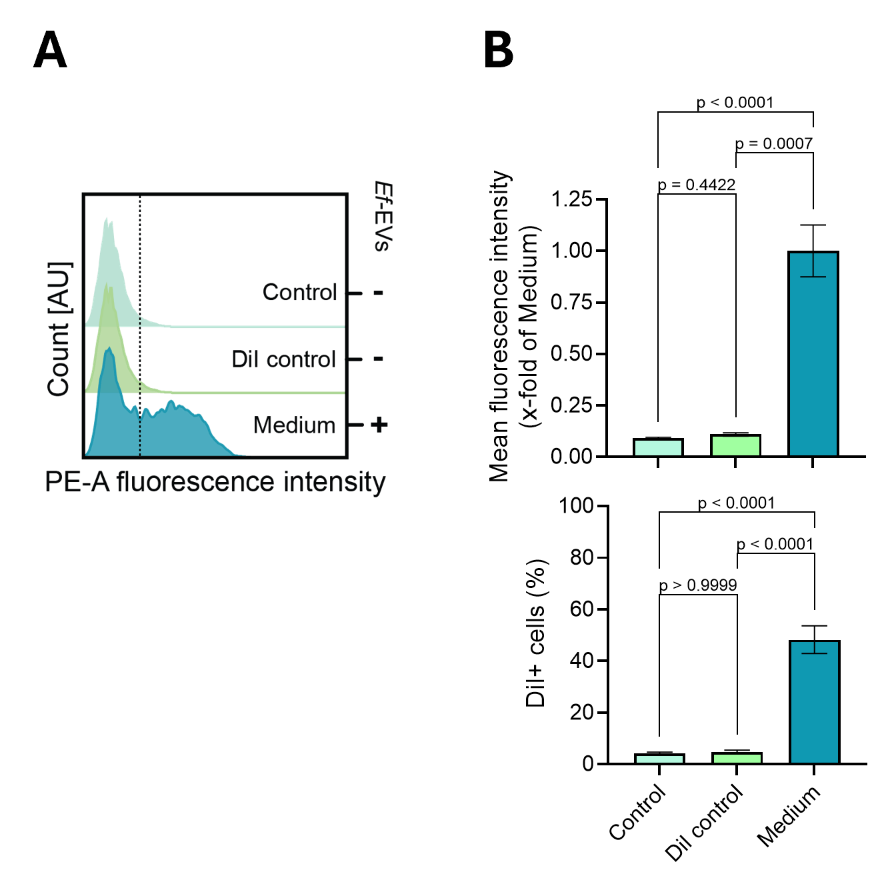


**Figure S5**: A and B) dTHP1-XBlue cells were treated with a control EV-mock solution (DiI control) consisting of SEC-fractionated DiI in PBS (final DiI concentration: 2 µM, DiI control) and DiI-labeled *Ef*-EVs (7000 EVs/cell). Cells incubated in cell culture medium alone were used as negative controls. Cells treated with cell culture medium supplemented with DiI-labeled *Ef*-EVs (7000 EVs/cell) were used as positive controls. Mean fluorescence intensity (B, upper panel) and EV-positive cells (B, lower panel) were quantified after 4 hours of treatment by measuring fluorescence intensity associated with DiI-labeled *Ef*-EVs on the PE channel. Results are presented as mean ± SD (N = 3, n = 3) and were analyzed by Kruskal-Wallis test followed by Dunn’s multiple comparison *post hoc* test


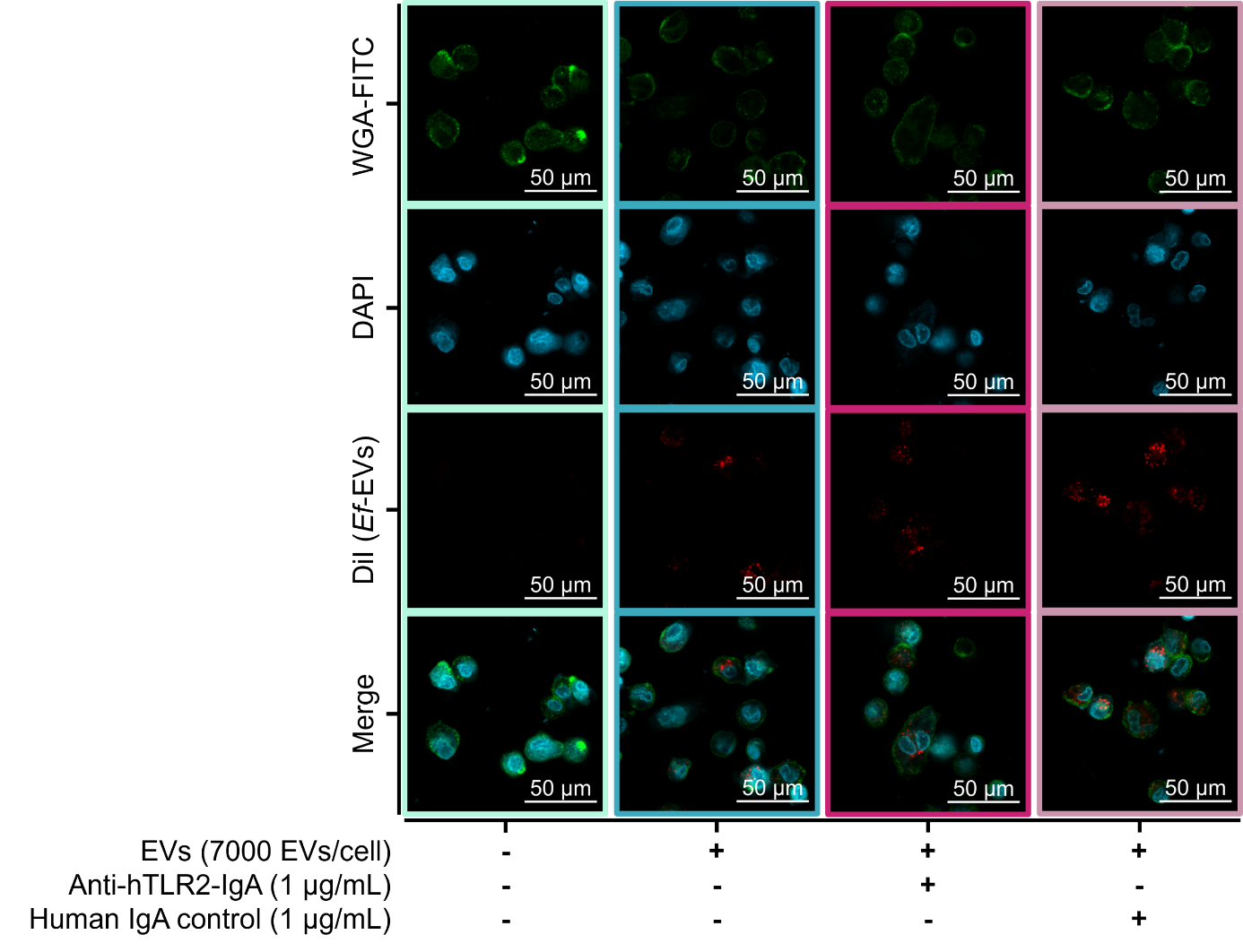


**Figure S6**: TLR2 does not function as an endocytic receptor for EV uptake. dTHP1-XBlue cells were pretreated with anti-hTLR2-IgA mAb (1 µg/mL) or human IgA2 control mAb (1 µg/mL) for 1 hour. Cells were then treated with *Ef*-EVs (7000 EVs/cell) in the presence of antibodies for 24 hours. Confocal micrographs present cell membrane (WGA-FITC panel), cell nuclei (DAPI panel), and fluorescence associated with DiI-labeled Ef-EVs (DiI panel) independently and merged (Merge panel) (scale bar = 50 µm).


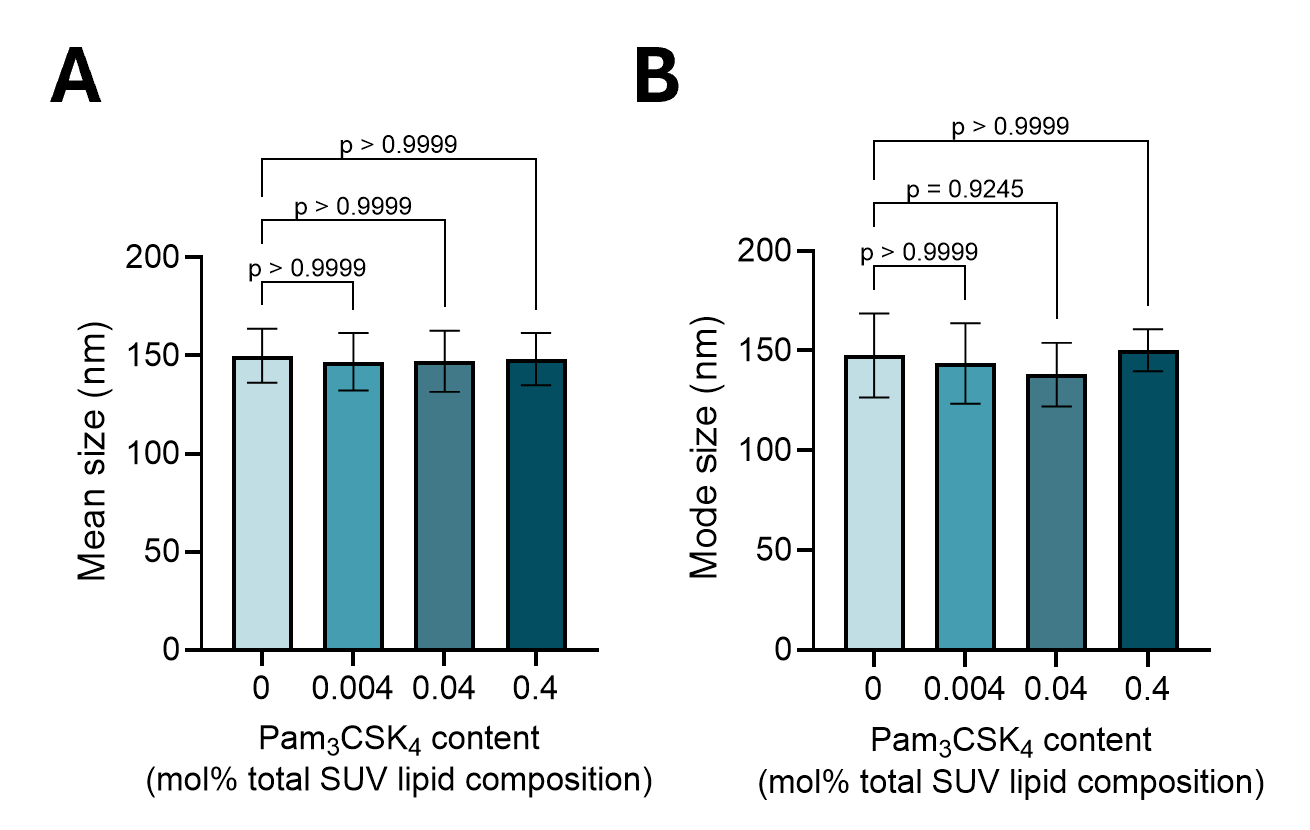


**Figure S7**: Particle size characterization of Pam_3_CSK_4_-SUVs as measured by NTA. A) Average mean size and B) average mode size of SUVs containing increasing amounts of Pam_3_CSK_4_ (from 0 to 0.4 mol% total SUV lipid composition). Results are presented as mean ± SD (N = 3, n = 3) and were analyzed by Kruskal-Wallis test followed by Dunn’s multiple comparison *post hoc* test.


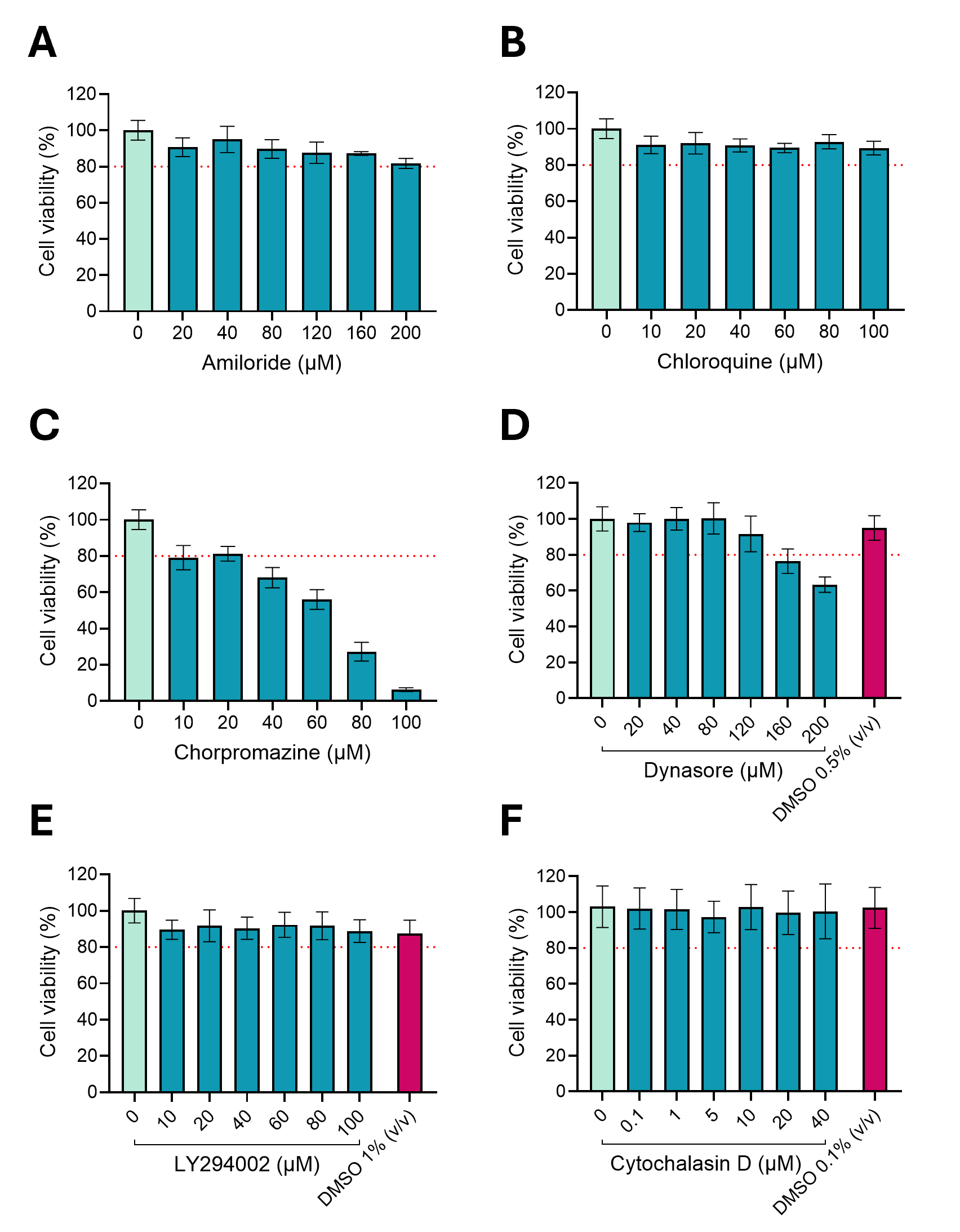


**Figure S8**: Toxicity screening of pharmacological inhibitors of endocytosis in dTHP1-XBlue cells. Cells were treated with different amounts of A) amiloride (macropinocytosis inhibitor, from 20 µM to 200 µM), B) chloroquine (clathrin-mediated endocytosis inhibitor, from 10 µM to 100 µM), C) chlorpromazine (clathrin-mediated endocytosis inhibitor, from 10 µM to 100 µM), D) dynasore (dynamin-dependent endocytosis inhibitor, from 20 µM to 200 µM), E) LY294002 (phagocytosis inhibitor, from 10 µM to 100 µM), and F) Cytochalasin (actin polymerization inhibitor, from 0.1 µM to 40 µM). After 4.5 hours of treatment, cell viability was assessed using the MTT assay. Results are shown as mean ± SD (N = 3, n = 3).


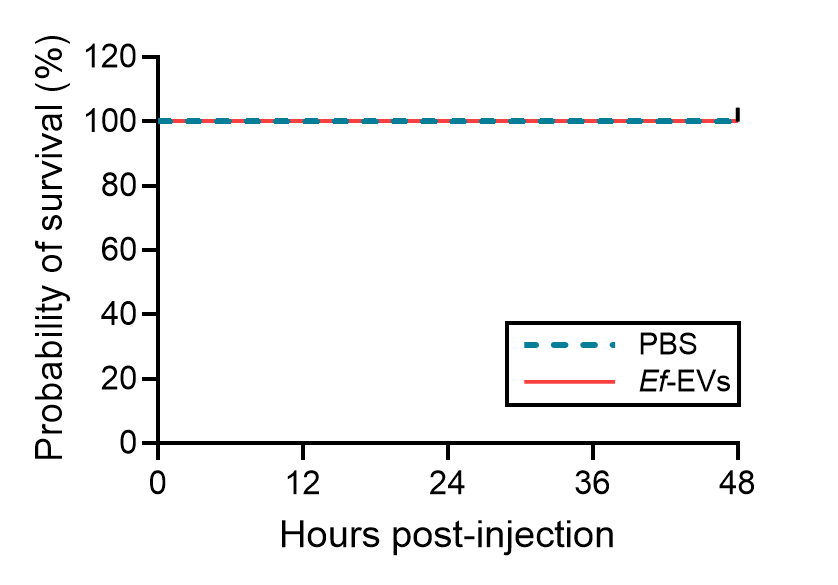


**Figure S9**: Kaplan-Meier graph shows the percentage of survival up to 48 hours post-injection with *Ef*-EVs and PBS. Zebrafish larvae were injected with either 4 nL *Ef*-EVs (200,000 EVs) or 4 nL PBS at the 3rd-dpf into the yolk sac (N = 3, n = 20). Results indicate the number of live larvae was monitored for 48 hours post-injection.


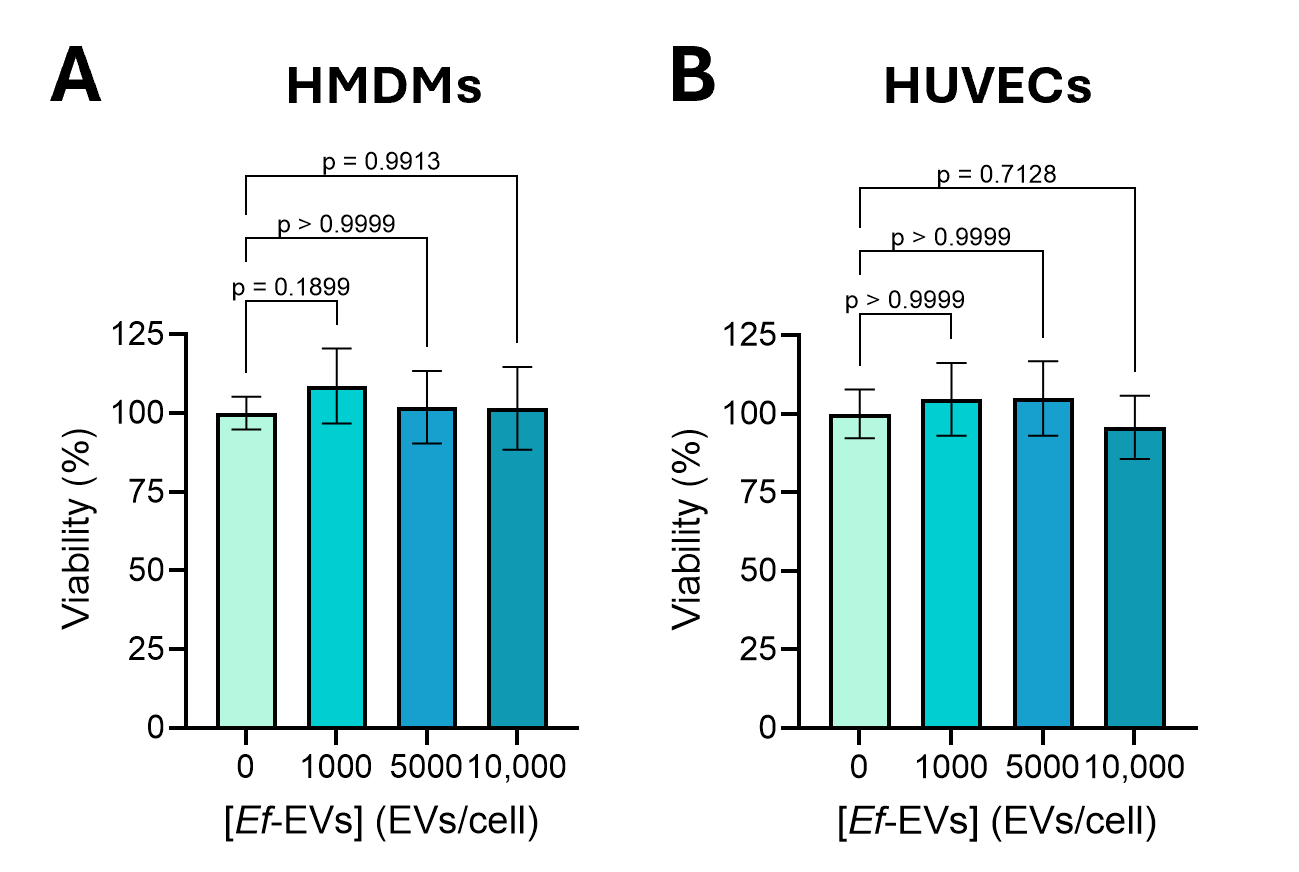


**Figure S10**: HMDMs (A) and HUVECs (B) were incubated with *Ef*-EVs (1000-10,000 EVs/cell). After 24 hours of incubation, cell viability was assessed using the MTT assay. Results are shown as mean ± SD of three individual donors (N = 3, n = 3) for HMDMs and mean ± SD of two individual donors (N = 2, n = 6) for HUVECs. Results were analyzed by Kruskal-Wallis test followed by Dunn’s multiple comparison *post hoc* test.
